# Development of a core outcome set for lateral elbow tendinopathy (COS-LET) using best available evidence and an international consensus process

**DOI:** 10.1136/bjsports-2021-105044

**Published:** 2022-02-08

**Authors:** Marcus Bateman, Jonathan P Evans, Viana Vuvan, Val Jones, Adam C Watts, Joideep Phadnis, Leanne M Bisset, Bill Vicenzino, COS-LET Authorship Group, Isabel Andia

**Affiliations:** 1 Orthopaedic Department, University Hospitals of Derby and Burton NHS Foundation Trust, Derby, UK; 2 Princess Elizabeth Orthopaedic Centre, Royal Devon and Exeter Hospital, Exeter, UK; 3 Health Services and Policy Research Group, University of Exeter Medical School, Exeter, UK; 4 School of Health & Rehabilitation Sciences: Physiotherapy, University of Queensland, Brisbane, Queensland, Australia; 5 Orthopaedic Department, Sheffield Teaching Hospitals NHS Foundation Trust, Sheffield, UK; 6 Upper Limb Unit, Wrightington Hospital, Wrightington, UK; 7 Department of Trauma and Orthopaedics, University Hospitals Sussex NHS Foundation Trust, Brighton, UK; 8 Brighton and Sussex Medical School, Brighton, UK; 9 Menzies Health Institute Queensland, Griffith University, Gold Coast, Queensland, Australia; 10 School of Health Sciences & Social Work, Griffith University, Gold Coast, Queensland, Australia

**Keywords:** elbow, tendinopathy

## Abstract

**Objectives:**

To develop a core outcome set for lateral elbow tendinopathy (COS-LET) and to provide guidance for outcome evaluation in future studies.

**Methods:**

We implemented a multi-stage mixed-methods design combining two systematic reviews, domain mapping of outcome measurement instruments to the core domains of tendinopathy, psychometric analysis of instruments, two patient focus groups and a Delphi study incorporating two surveys and an international consensus meeting. Following the OMERACT guidelines, we used a 70% threshold for consensus.

**Results:**

38 clinicians/researchers and 9 patients participated. 60 instruments were assessed for inclusion. The only instrument that was recommended for the COS-LET was Patient Rated Tennis Elbow Evaluation (PRTEE) for the disability domain. Interim recommendations were made to use: the PRTEE function subscale for the function domain; PRTEE pain subscale items 1, 4 and 5 for the pain over a specified time domain; pain-free grip strength for the physical function capacity domain; a Numerical Rating Scale measuring pain on gripping for the pain on activity/loading domain; and time off work for the participation in life activities domain. No recommendations could be made for the quality-of-life, patient rating of condition and psychological factors domains.

**Conclusions:**

The COS-LET comprises the PRTEE for the disability domain. Interim-use recommendations included PRTEE subscales, time off work, pain-free grip strength and a Numerical Rating Scale measuring pain on gripping. Further work is required to validate these interim measures and develop suitable measures to capture the other domains.

## Introduction

### Background and objectives

Pain arising from the tendons on the lateral side of the elbow is common in adults, particularly in middle age.^1^ Historically, it has been known by various names such as lateral epicondylitis or tennis elbow, but the current accepted description is lateral elbow tendinopathy (LET).^2^ It is acknowledged that there is substantial heterogeneity of outcome measure instrument use in elbow research and specifically for LET.^3^ With no clear consensus on which instruments most accurately represent a patient’s LET-related health status, comparison of effectiveness research and evidence synthesis/meta-analysis has been hampered.

In 2019, an international group of experts in the field of tendinopathy (International Scientific Tendinopathy Symposium Consensus (ICON) Group), comprising researchers, healthcare professionals and patients, published a consensus document defining the nine health-related core domains of tendinopathy. That group recommended researchers and clinicians measure outcomes for specific regional tendinopathies against these domains.^4^

The aim of this project was to develop a core outcome set (COS) for LET that maps to the nine domains. A COS is a minimal set of outcome measures to be used in future research and clinical practice involving people with LET. It enables meta-analysis of findings from different studies using a consistent set of measures. To be included in a COS, measures need to be both practical to perform (based on cost, patient burden and availability) and of high quality (valid, responsive, reliable, interpretable and of acceptable burden for patients and investigators).^5^ The result will be a minimum set of outcome measurement instruments to be used in future LET research that allows direct comparison between different studies across the nine domains.

### Scope

This COS relates to all adults diagnosed with LET and applies to interventional research (including surgical and non-surgical) and longitudinal assessment. The COS will only apply to the English language.

## Method

We designed the project following the COSMIN–COMET guideline.^6^ We developed a COS that was based on a consensus of perspectives gained from healthcare professionals with expertise in LET and patients with the condition. This involved a multi-stage stepwise process, which started with identifying the instruments used in studies of LET by updating a previous systematic evaluation of patient-rated outcomes for LET.^7^ These instruments were then mapped by the steering committee to the nine core tendinopathy domains.^4^ The mapped outcome measurement instruments were then subjected to the OMERACT truth (part a) and feasibility filters^8^ by participants in the first round of a Delphi survey. We (MB and JPE) then systematically evaluated the psychometric properties of the included instruments—applying the OMERACT truth (part b) and discrimination filters,^8^ using the EMPRO tool.^9^ This information then formed the basis of the second Delphi survey, which was conducted to make recommendations for a COS-LET. Focus groups were conducted with patients to review findings after Delphi survey 2. The results of the surveys and focus groups were then reviewed and discussed by participants at an international consensus online meeting (Delphi stage 3), before voting to determine the final COS-LET. The study was led by an international eight-person steering committee with expertise in LET—comprising a mix of junior and senior researchers and clinicians from surgical and physiotherapy backgrounds.

### Protocol/ registry entry

We registered the project with the Core Outcome Measures in Effectiveness Trials Initiative (http://www.comet-initiative.org/Studies/Details/1497) and published the protocol in an *open access journal.* (https://trialsjournal.biomedcentral.com/articles/10.1186/s13063-021-05291-9). This report follows the COS Standards for Reporting checklist.^10^

### Participants

The Delphi study population comprised of experienced clinicians and researchers nominated by the steering committee, identified by their reputation as elbow clinical specialists or prior publications related to LET. Additionally, a search of the Expertscape^11^ and SCOPUS^12^ databases by author and filtered by the terms ‘tennis elbow’ and ‘trial’ identified a list of other researchers to approach. Representation from a range of nationalities, with a spread of ethnicity and sex was ensured.

Patient representatives were invited by the clinicians on the steering committee.

### Information sources

**Figure 1:**
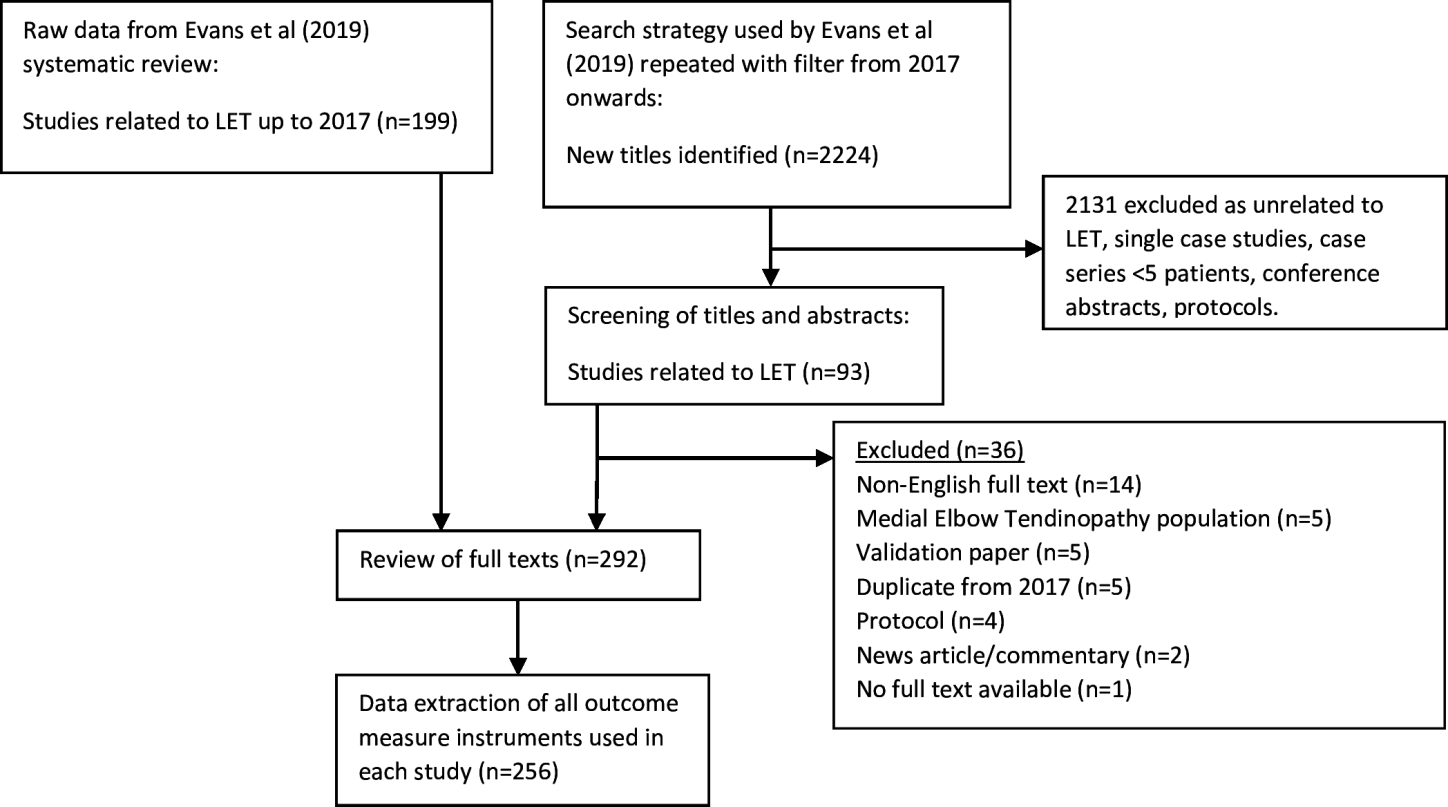
Adapted PRISMA flowchart: to review the outcome measure instruments used in all LET studies. LET, lateral elbow tendinopathy; PRISMA, Preferred Reporting Items for Systematic Reviews and Meta-Analyses.

In order to comprehensively evaluate all outcome measurement instruments used in research of LET, we systematically reviewed the literature. To do this, we updated the 2019, Evans *et al*,^7^ systematic review of English language instruments used in surgical and non-surgical trials for LET (census date: 1 May 2017). The search results were screened initially by title and abstract by two reviewers (MB and JPE) independently of each other using the online Rayyan tool^13^—any disagreements were discussed and reconciled. We included all study designs except research protocols, case studies and small case series of less than five patients. One hundred and ninety nine full texts from the original search and 93 from the updated searches (to February 2020) were screened down to 256 papers for data extraction—providing a comprehensive list of instruments used in LET research (figure 1). Extracted data included all outcome instruments used, number of patients included in the study and full details of any novel instruments.

### Consensus process and outcome scoring

The retrieved instruments were then submitted to a stepwise consensus process that mapped them to the core tendinopathy domains. The mapped instruments where then used to construct the first survey. The instruments agreed to in that survey were then evaluated for their psychometrics—the results of which were included in a second survey. Results of the surveys were discussed in two patient focus groups. Finally, a consensus meeting reviewed and discussed findings before voting on the final COS-LET.

Instruments mapped to domains: the steering committee members mapped each instrument to the nine core tendinopathy domains^4^: patient rating of condition; participation in life activities (day to day, work and sport); pain on activity/loading; function; psychological factors; physical function capacity; disability; quality of life; and pain over a specified time. Each instrument was mapped by two steering committee members independently, then compared and reconciled if needed—using the instrument’s published development study or manual.

Survey 1: each instrument and its reference document, including original development study and/or manual, were presented per mapped domain to participants in the first online survey using Qualtrics (Provo, Utah, USA). Participants were asked to respond yes/no/unsure whether each instrument was a truthful measure of the domain (valid), feasible to use clinically and whether it should be included in the COS-LET.

Outcomes were scored using the OMERACT traffic light system,^8^ whereby responses achieving <30% agreement were rated red and excluded; those achieving ≥70% were rated green and included; and those achieving 30%–69% were rated amber, inconclusive but not excluded.

**Figure 2:**
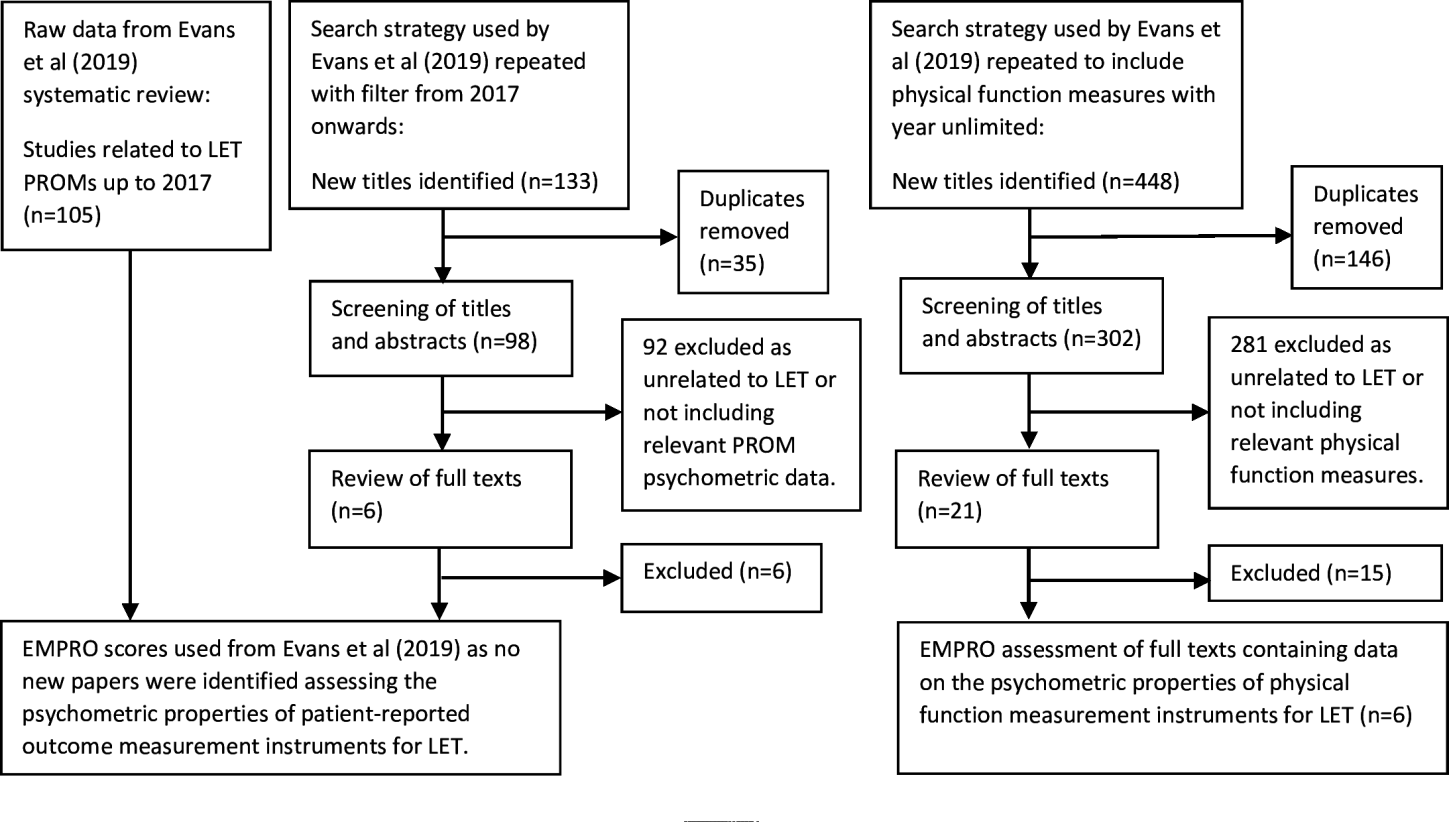
Adapted PRISMA flowchart: to review the psychometric properties of the instruments included after Delphi round 1. LET, lateral elbow tendinopathy; PRISMA, Preferred Reporting Items for Systematic Reviews and Meta-Analyses; PROMs, Patient-Reported Outcome Measures.

Psychometric evaluation of instruments: following exclusion of instruments (<30% agreement in survey 1) and inclusion of any new instruments proposed by respondents, the OMERACT truth (part b) and discrimination filters were applied by two members of the steering committee (MB and JPE). This involved an update of Evans *et al*’s^7^ systematic review to identify instrument development or validation studies (figure 2). These members used the EMPRO tool, separately to each other, to assess the psychometric properties (construct validity, reliability, repeatability, responsiveness and interpretability) of each instrument, then meeting to discuss points of contention.^9^ These were resolved without the need of a third assessor. The steering committee then voted anonymously, using the OMERACT traffic light system, on whether each instrument should be considered ‘Good to go’, ‘A concern/more work needed’ or ‘Stop, do not continue’.^8^ This voting stage was included to ratify the psychometric evaluation process and was guided by an EMPRO score threshold of 40% for inclusion.

Survey 2: during the second Delphi survey, participants were presented with the findings of Delphi survey 1 (online supplemental file 1), showing the traffic light rating of each instrument within their associated matched domain, and subsequent outcome of the truth (part b) and discrimination filters (online supplemental file 2). Participants were asked to rate instruments that achieved a nominal EMPRO score of ≥40% for inclusion in the final COS-LET (yes/no/unsure). Those instruments that were no or unsure for the final COS-LET, had no psychometric data or had an EMPRO score of <40% were rated for interim use (yes/no). The responses were analysed and those instruments achieving <30% of votes were excluded.

10.1136/bjsports-2021-105044.supp1 Supplementary data

10.1136/bjsports-2021-105044.supp2 Supplementary data

Patient focus groups: results of the Delphi stages, inclusive of survey 2, were then discussed at an online patient focus group for UK patients and another for Australian patients. Patients were asked to provide their insights/perspectives on the decisions to date and to ratify any instruments voted ≥70%.

Final consensus meeting: participants attended an online consensus meeting to discuss the findings of the Delphi process to date (including patient focus group outcomes) and to vote on outcome measures in the COS-LET and for interim use. A report of the results of previous surveys and patient focus groups (online supplemental file 3) was provided to the participants 2 weeks prior.

10.1136/bjsports-2021-105044.supp3 Supplementary data

### Consensus definition

For each domain, instruments voted for by ≥70% of participants in both surveys and at the meeting were included in the COS-LET. For domains where no instruments were agreed, interim suggestions were proposed based on a green light from Delphi survey 1 (≥70%), and amber light from Delphi survey 2 (30%–69%) and ≥70% agreement from the consensus meeting vote.

## Results

We commenced this study in January 2020, with regular steering committee working meetings to plan and design data collection. Data collection was completed at the consensus meeting on 5 May 2021.

### Protocol deviations

The only deviation from the published protocol was that patient focus groups were conducted, rather than one-to-one interviews. This decision was taken to allow for patient interaction and group discussion, the impact of which on our findings is likely low to negligible.

### Participants

We invited 58 healthcare professionals of which 40 agreed to participate, 7 did not agree (retired (2) and no reason given (5)) and 11 did not respond (maternity leave out of office message (1) and unknown reason (10)). Thirty eight engaged with the process and 2 withdrew. Thirty six (90%) of the clinicians/researchers who agreed to participate fully completed both surveys (table 1), 2 (5%) completed one survey and 31 (84%) of those completing surveys attended the online meeting. The clinician/researcher cohort consisted mainly of physiotherapists or orthopaedic surgeons, located in Europe or Australia, and had research higher degree training.

**Table 1:** Participant characteristics (n (%) unless otherwise stated)

Characteristics	Clinicians/researchers			Patients
	Survey 1 (n=37)	Survey 2* (n=37)	Meeting (n=31)	Survey 1/focus group (n=9)
Sex: male	25 (67.6)	25 (67.6)	22 (71.0)	4 (44.4)
Age: median (IQR; minimum–maximum), years	51 (43–57; 34–68)	51 (43–55; 34–68)	51 (43–53; 34–68)	48 (37–53; 26–59)
Role				
Clinician	2 (5.4)	2 (5.4)	2 (6.4)	NA
Researcher	5 (13.5)	5 (13.5)	4 (12.9)	NA
Clinician researcher	30 (81.1)	30 (81.1)	25 (80.7)	NA
Highest academic qualification				
PhD	21 (56.8)	21 (56.8)	17 (54.8)	–
Master	6 (16.2)	6 (16.2)	5 (16.1)	2 (22.2)
Doctor of Medicine	7 (18.9)	7 (18.9)	7 (22.6)	–
Postgraduate diploma/certificate	–	–	–	2 (22.2)
Bachelor	3 (8.1)	3 (8.1)	2 (6.5)	4 (44.4)
No university qualification	–	–	–	1 (11.1)
Profession				
Physiotherapy	16 (43.2)	16 (43.2)	14 (45.2)	NA
Orthopaedic surgeon	14 (37.8)	14 (37.8)	12 (38.7)	NA
Sports and exercise medicine physician	3 (8.1)	3 (8.1)	2 (6.4)	NA
Not specified	3 (8.1)	3 (8.1)	2 (6.4)	NA
Rheumatologist	1 (2.7)	1 (2.7)	1 (3.2)	NA
Therapy radiographer	–	–	–	1 (11.1)
Health information technology	–	–	–	1 (11.1)
Non-healthcare professional	–	–	–	7 (77.8)
Lateral elbow tendinopathy				
Current case	1 (2.7)	1 (2.7)	1 (3.2)	5 (55.6)
History	10 (27.0)	11 (29.7)	9 (29.0)	6 (66.7)
Country where work†				
Europe	20 (54.1)	20 (54.1)	16 (51.2)	5 (55.6)
Australia	11 (29.7)	10 (27.0)	8 (25.8)	4 (44.4)
North America	5 (13.5)	5 (13.5)	5 (16.1)	–
Asia	1 (2.7)	2 (5.4)	2 (6.5)	–

*1 person from Australia did survey 1 but not 2; another did survey 2 not 1 (technical issues).

†Countries grouped per continent as follows: Europe=Belgium, Finland, Greece, Italy, the Netherlands, Norway, Spain, Sweden, Turkey (Istanbul) and UK; North America=Canada and USA; and Asia=India and Israel.

Nine patients from the UK and Australia participated in the study—7 completed the first survey and 5 participated in the focus groups.

### Outcomes

Sixty unique instruments were identified from the first systematic review and included in survey 1 (table 2). From survey 1, three measures: pain on gripping, Patient Rated Tennis Elbow Evaluation (PRTEE)^14–16^ and Quick Disability of Arm, Shoulder and Hand Questionnaire (DASH),^17^ were the only ones reaching ≥70% for both the patient and the clinician/researcher groups. The Patient Specific Function Scale^18^ (function domain) and Tennis Elbow Functional Scale^19^ (both pain domains) were voted to be in the COS-LET by patients who commented favourably on the scores’ item level face validity—however, the lack of robust psychometric evaluation in LET populations precluded their inclusion following clinician/researcher evaluation (table 2). Twenty four instruments were excluded after this survey as they received <30% of votes for inclusion in the COS-LET by both patients and clinicians/researchers. Participants proposed an additional seven instruments (table 2).

**Table 2:** Showing the prevalence of use of individual instruments (shown as the number of previous studies and %) and responses to Delphi survey 1 (shown as the number of responses and %)

Domain: measure	Prevalence	Clinicians and researchers				Patients (n=7)		
		*n	a	b	c	a	b	c
Patient rating of condition								
Global Perceived Effect Score	1 (<1)	39	22 (56)	28 (72)	36 (92)	5 (71)	6 (86)	6 (86)
GROC	9 (4)	39	25 (64)	29 (74)	34 (87)	4 (57)	6 (86)	6 (86)
Patient Satisfaction Scale	29 (11)	39	20 (51)	25 (64)	35 (90)	5 (71)	6 (86)	6 (86)
Roles and Maudsley†	15 (6)							
Participation (daily activities, work and sport)								
Return to sport	5 (2)	39	15 (38)	25 (64)	32 (82)	5 (71)	5 (71)	6 (86)
Time off work	16 (6)	39	21 (54)	31 (79)	30 (77)	4 (57)	4 (57)	6 (86)
Total Elbow Scoring System	1 (<1)	39	12 (31)	14 (36)	21 (54)	4 (57)	4 (57)	5 (71)
OSTRC question 1†	0							
Pain on activity/loading								
Tennis Elbow Functional Scale	2 (1)	39	11 (28)	24 (62)	22 (56)	6 (86)	6 (86)	6 (86)
Thomsen Test (VAS pain resisted wrist extension)	7 (3)	39	14 (36)	28 (72)	28 (72)	5 (71)	5 (71)	6 (86)
VAS chair pick-up	2 (1)	39	9 (23)	20 (51)	23 (59)	2 (29)	6 (86)	4 (57)
VAS pain during activity	49 (19)	39	31 (79)	35 (90)	34 (87)	4 (57)	5 (71)	6 (86)
VAS pain during elbow movement	5 (2)	39	7 (18)	13 (33)	27 (69)	2 (29)	3 (43)	6 (86)
VAS pain on gripping	16 (6)	39	28 (72)	37 (95)	35 (90)	5 (71)	5 (71)	7 (100)
VAS pain at work	11 (4)	39	15 (38)	23 (59)	29 (74)	3 (43)	3 (43)	4 (57)
Pain Free Functional Index	2 (1)	39	7 (18)	20 (51)	23 (59)	3 (43)	3 (43)	6 (86)
PRTEE†‡	78 (30)	38	24 (63)	30 (79)	27 (71)	7 (100)	7 (100)	7 (100)
Function								
Patient Specific Functional Scale	1 (<1)	38	11 (29)	24 (63)	20 (53)	5 (71)	6 (86)	6 (86)
Upper Extremity Functional Scale	5 (2)	38	17 (45)	26 (68)	26 (68)	4 (57)	4 (57)	7 (100)
VAS function	6 (2)	38	17 (45)	24 (63)	29 (76)	2 (29)	3 (43)	4 (57)
PRTEE†‡	78 (30)							
Psychological factors								
Hospital Anxiety and Depression Scale	3 (1)	38	14 (37)	20 (53)	19 (50)	2 (29)	2 (29)	6 (86)
Tampa Scale of Kinesophobia	4 (2)	38	15 (39)	19 (50)	21 (55)	5 (71)	6 (86)	5 (71)
STAI trait†	0							
Nottingham Health Profile†	3 (1)							
Physical function capacity (eg, strength)								
Grip strength (maximum)	93 (36)	38	18 (47)	31 (82)	26 (68)	6 (86)	6 (86)	7 (100)
Pain-free grip strength	42 (16)	38	25 (66)	30 (79)	27 (71)	6 (86)	6 (86)	7 (100)
Elbow ROM	6 (2)	38	4 (11)	12 (32)	25 (66)	3 (43)	2 (29)	7 (100)
Disability								
Andrews-Carson	2 (1)	38	0 (0)	3 (8)	10 (26)	1 (14)	1 (14)	3 (43)
ASES score	5 (2)	38	4 (11)	17 (45)	13 (34)	3 (43)	3 (43)	5 (71)
Broberg and Morrey Rating System	1 (<1)	38	1 (3)	8 (21)	10 (26)	2 (29)	3 (43)	5 (71)
DASH	77 (30)	38	15 (39)	26 (68)	17 (45)	4 (57)	4 (57)	5 (71)
HAND10	1 (<1)	38	3 (8)	17 (45)	23 (61)	4 (57)	4 (57)	6 (86)
Japanese Orthopaedic Association Elbow Score	2 (1)	38	4 (11)	17 (45)	16 (42)	0 (0)	0 (0)	4 (57)
Laitinen Questionnaire	1 (<1)	38	5 (13)	14 (37)	15 (39)	1 (14)	1 (14)	4 (57)
Liverpool Elbow Score	1 (<1)	38	1 (3)	13 (34)	13 (34)	2 (29)	3 (43)	3 (43)
Mayo Elbow Performance Score	30 (12)	38	3 (8)	12 (32)	15 (39)	0 (0)	0 (0)	2 (29)
Nirschl	18 (7)	38	7 (18)	22 (58)	21 (55)	4 (57)	4 (57)	4 (57)
Nottingham Health Profile	3 (1)	38	1 (3)	6 (16)	12 (32)	0 (0)	0 (0)	0 (0)
Oxford Elbow Score	4 (2)	38	9 (24)	20 (53)	21 (55)	4 (57)	5 (71)	6 (86)
Patient-Rated Wrist Evaluation Questionnaire	1 (<1)	38	5 (13)	17 (45)	19 (50)	4 (57)	4 (57)	6 (86)
PRTEE†‡	78 (30)	37	27 (73)	34 (92)	32 (86)	6 (86)	6 (86)	6 (86)
Quick DASH	37 (14)	38	27 (71)	29 (76)	31 (82)	7 (100)	7 (100)	7 (100)
Total Elbow Scoring System	1 (<1)	38	4 (11)	14 (37)	20 (53)	2 (29)	2 (29)	4 (57)
Roles and Maudsley	15 (6)	38	2 (5)	11 (29)	22 (58)	1 (14)	2 (29)	4 (57)
Quality of life								
EQ5D	9 (4)	37	19 (51)	22 (59)	25 (68)	4 (57)	4 (57)	6 (86)
SF-36	13 (5)	37	6 (16)	23 (62)	9 (24)	1 (14)	5 (71)	2 (29)
SF-12	4 (2)	37	15 (41)	25 (68)	25 (68)	3 (43)	5 (71)	4 (57)
Nottingham Health Profile†	3 (1)							
WHOQol BREF†	0							
SMFA†	0							
Pain over a specified timeframe								
VAS night pain	9 (4)	37	14 (38)	26 (70)	32 (86)	2 (29)	4 (57)	6 (86)
VAS pain defined time period	22 (9)	37	25 (68)	30 (81)	29 (78)	2 (29)	3 (43)	5 (71)
VAS pain at rest	52 (20)	37	21 (57)	25 (68)	31 (84)	1 (14)	2 (29)	5 (71)
Tennis Elbow Functional Scale	2 (1)	37	8 (22)	22 (59)	21 (57)	6 (86)	6 (86)	6 (86)
Measures from review not mapped								
Analgesic use	2 (1)	37	16 (43)			1 (14)		
Canadian Occupational Performance Measure	1 (<1)	37	2 (5)			1 (14)		
Cold Pain Threshold	5 (2)	37	3 (8)			1 (14)		
EMG	5 (2)	37	0 (0)			2 (29)		
Gothenburg QoL Instrument	1 (<1)	37	0 (0)			0 (0)		
MRI appearance	5 (2)	37	3 (8)			1 (14)		
ORI-TETS	3 (1)	37	1 (3)			1 (14)		
Placzek Score	1 (<1)	37	4 (11)			2 (29)		
Pressure Pain Threshold	24 (9)	37	7 (19)			1 (14)		
US appearance	13 (5)	37	4 (11)			1 (14)		
UoP-PFQ	2 (1)	37	1 (3)			1 (14)		
VAS pain on palpation	12 (5)	37	4 (11)			2 (29)		
VAS pain overall	101 (39)	37	9 (24)			2 (29)		
WL-26	1 (<1)	37	2 (5)			4 (57)		

a: this measure should be in the core outcome set for lateral elbow tendinopathy.

b: this measure has content and face validity.

c: the measure is clinically feasible.

Green (yes responses: ≥70%) and amber (unsure responses: 30%–69%) were progressed to subsequent stages of the Delphi, whereas red (<30%) were removed.

*2 participants partially completed the first survey and their data was included herein.

†Outcome measures suggested by participants, noting PRTEE was not new to the list.

‡Removal of one participant’s response who was designer of the measure.

ASES, American Shoulder and Elbow Surgeons; DASH, Disability of Arm, Shoulder and Hand Questionnaire; EMG, electromyography; GROC, Global Rating of Change; ORI-TETS, Orthopaedic Research Institute-Tennis Elbow Testing System; OSTRC, Oslo Sport Trauma Research Centre Questionnaire; PRTEE, Patient Rated Tennis Elbow Evaluation; QoL, quality of life; ROM, range of movement; SF-12, 12-item Short Form Health Survey; SF-36, 36-item Short Form Health Survey; SMFA, Short Musculoskeletal Function Assessment; STAI, State–Trait Anxiety Inventory; UoP-PFQ, University of Peloponnese Pain, Functionality and Quality of Life Questionnaire; US, ultrasound; VAS, Visual Analogue Scale; WHOQol BREF, WHO Quality of Life Abbreviated Questionnaire; WL-26, Work Limitation 26-item Questionnaire.

#### Core outcome set

A search for studies of the measures with ≥30% responses from survey 1 revealed that only 8/21 (38%) instruments had been evaluated for their psychometric properties in specific LET populations. These measures were submitted to analysis with the EMPRO tool—scoring between 25% and 73% (table 3). The additional instruments proposed by responders in survey 1 had no psychometric data for the LET population and were not considered further in the development of the COS-LET. Seven instruments had an EMPRO score of ≥40 and received ≥70% of votes in survey 1 from either patients or clinicians/researchers—they were: DASH,^17 20–22^ Quick DASH,^17 23 24^ Oxford Elbow Score,^25 26^ PRTEE,^14–16^ Tennis Elbow Functional Scale,^19^ as well as grip strength.^27–29^ These were then independently assessed by the steering committee using the OMERACT truth (part b) and discrimination filters (results given in online supplemental file 2), which along with results from survey 1, were available to inform clinician’s/researcher’s decisions in survey 2. The results of survey 2 are presented in table 3—only the PRTEE met the threshold for inclusion in the COS-LET for the disability domain, which was ratified at the consensus meeting.

**Table 3:** Results of the clinimetric evaluation (EMPRO), second survey, patient focus group and final consensus meeting decision arranged in reverse chronology across the table—for each measure per domain. Data are frequency count (%) unless otherwise specified. The COS-LET is in ‘Part A’ and the interim suggestions of measures that might be used and studied are in ‘Part B’

Part A: core outcome to be used in clinical trials and cohort studies									
Domain	Measure	Decision	Votes	Patient	Survey 2				EMPRO
			Yes*	Agreed	In	Out	Unsure	Interim	Score %
Disability	PRTEE	√	29 (100)	√	26 (70.3)	5 (13.5)	6 (16)	n/a	57.0
	DASH	x			3 (8.1)	25 (67.6)	9 (24.3)	n/a	66.9
	Quick DASH	x			22 (59.5)	9 (24.3)	6 (16.2)	n/a	72.5
	Oxford Elbow Score	x			6 (16.2)	19 (51.4)	12 (32.4)	n/a	66.6
**Part B: interim suggestion for use in clinical trials and as a focus of future clinimetric research**									
**Domain**	**Measure**	**Decision**	**Votes**	**Patient**	**Survey 2**				**EMPRO**
			Yes*	**Agreed**	**In**	**Out**	**Unsure**	**Interim**	**Score %**
Function	PRTEE—relevant items	√	30 (96.8)	√	24 (64.9)	5 (13.5)	8 (21.6)	33 (89.2)	n/a
Pain over specified time	PRTEE pain subscale items 1, 4 and 5†	√	30 (96.8)						n/a
	Tennis Elbow Functional Scale	x	n/a		3 (8.1)	27 (73)	7 (18.9)	9 (24.3)	41.7
Physical function capacity	Pain-free grip strength	√	26 (86.7)	√	15 (40.5)	11 (29.7)	11 (29.7)	24 (64.9)	32.9
	Maximum grip strength	x	n/a		6 (16.2)	17 (46)	14 (37.8)	12 (32.4)	25.1
Pain on loading/activity	Pain on gripping†	√	25 (83.3)		n/a	n/a	n/a	n/a	n/a
	Tennis Elbow Functional Scale	x	–		4 (10.8)	27 (73)	6 (16.2)	7 (18.9)	41.7
	PRTEE pain subscale items 2 and 3	x	19 (65.5)	√	24 (64.9)	7 (18.9)	6 (16.2)	31 (83.8)	n/a
Participation	Time off work	√	22 (73.3)		n/a	n/a	n/a	26 (70.3)	n/a
	Time off sport	x	18 (60)		n/a	n/a	n/a	21 (56.8)	n/a
QoL	EQ5D	x	20 (69)		n/a	n/a	n/a	22 (59.5)	n/a
	SF-12	x	6 (20.7)		n/a	n/a	n/a	14 (37.8)	n/a
Participant Rating of Condition	GROC	x	20 (66.7)	√‡	n/a	n/a	n/a	21 (56.8)	n/a
	Global Perceived Effect Score	x	n/a		n/a	n/a	n/a	13 (35.1)	n/a
	Patient Satisfaction Scale	x	n/a		n/a	n/a	n/a	17 (46)	n/a
Psychological	Tampa Scale of Kinesiophobia	x	10 (34.5)	√‡	n/a	n/a	n/a	16 (43.2)	n/a
	Hospital Anxiety and Depression Scale	x	n/a		n/a	n/a	n/a	14 (37.8)	n/a

*Note that not all 31 attendees voted on all items (at least, 29 voted on 4 items)—due to time zone differences. See appendices for full data.

†Pain on gripping was voted in survey 1, had no clinimetric evidence but was strongly supported for interim use—noting there were 2 (6.7%) participants unsure.

‡Patients were asked for their opinions on which of the measures most closely measured their condition domain and was feasible clinically.

COS-LET, core outcome set for lateral elbow tendinopathy; DASH, Disabilities of the Arm, Shoulder and Hand Questionnaire; GROC, Global Rating of Change; PRTEE, Patient Rated Tennis Elbow Evaluation; QoL, quality of life; SF-12, 12-item Short Form Health Survey.

### Interim recommendations

Where there was no measure in a domain that met the criteria for the COS-LET, we considered measures that reached ≥70% hurdle in survey 1—aiming to recommend one per domain to be used in the interim and as a direction for future research (table 3, Part B).

Of the measures that had psychometric data—that is, Tennis Elbow Functional Scale, maximum grip strength, pain-free grip strength and the PRTEE pain and function subscales—only the latter was voted as an interim measure in survey 2 (table 3). The patients (focus groups) agreed that items/subscales of the PRTEE (for pain on loading/activity, function and pain over specified time domains) and pain-free grip strength (for physical function capacity domain) were relevant to their condition. The consensus meeting decided that relevant subscale items from the PRTEE would be recommended as interim measures for function and pain over specified time domains. Pain-free grip strength was selected over maximum grip strength as it was thought to be more clinically and patient relevant. The meeting decided that pain on gripping, which had support in survey 1 but with no psychometric data, would be the preferred measure for pain on loading/activity domain, instead of the relevant subscale items from the PRTEE (table 3).

Of the other measures that did not have psychometric data (see table 3), only time off work was voted as an interim measure in survey 2—it was ratified at the consensus meeting.

Notably, after the consensus meeting, there were three domains for which no interim measures were agreed. These were: quality of life, participant overall rating of condition and psychological.

## Discussion

This is the first attempt to determine the minimum COS-LET. We reached agreement for an outcome measure for one of the nine tendinopathy domains—PRTEE for disability. Although the PRTEE has been found to be psychometrically robust, we note that the total EMPRO score was lower than other measures considered for inclusion. As previously identified, as the PRTEE was developed without patient involvement, its EMPRO score is, therefore, reduced as a consequence.^7^ However, the expert and patient groups within this COS development were concordant in their agreement that the PRTEE preferentially aligned with the disability domain. It was not possible to include measures in a COS-LET for the remaining domains, because there was either no instrument or a lack of instrument validation. In the interim, we decided on measures to recommend for validation studies and use in trials of LET.

The PRTEE function subscale was selected as an interim measure for the function domain, but concerns were raised that it mainly queries basic tasks and not higher-level tasks required in sports. This requires further investigation, along with the psychometric properties of the subscale. Patient responses from survey 1 favoured the Patient Specific Functional Scale for the function domain, but this was not supported by clinicians/researchers. This scale can be tailored to athletic/high-level tasks so may be an area for future investigation.

The pain over a specified time domain had no suitable instruments following survey 2. We resolved at the consensus meeting to recommend three questions from the PRTEE (pain at rest, least and worst over the last week) in the interim. The PRTEE had already been accepted for the COS-LET, thereby minimising patient burden—a key priority identified in the patient focus groups; however, the psychometric properties of these PRTEE items require further assessment.

Pain-free grip strength was recommended as an interim measure for physical function capacity domain, but it was not included in the final COS-LET due to limited validation in LET populations. With clear stakeholder approval, further validation work should be prioritised.

Measuring the pain on activity/loading domain was the source of a lengthy discussion, because the two options with sufficient psychometric evidence failed to reach consensus. Discussion then moved to rating pain on gripping as an interim measure. Gripping was raised as a common pain provoking activity in the patient focus groups. It had been voted in survey 1 to be in the COS-LET by patients and clinicians/researchers, but due to a lack of research it was not voted in survey 2. In the meeting, concerns were raised about standardising the gripping task—as this would be difficult across sexes and different work/sport contexts. We resolved to recommend using a Numerical Rating Scale to record pain during gripping in the interim and to prioritise its validation.

Time off work was recommended as an interim measure of the participation domain, but there were concerns regarding the definition of work and whether this was applicable to patients who were retired, unemployed, students or full-time parents/carers. Due to the value of using time off work as part of health economic evaluation, however, it was agreed that it should still be used in the interim. It was recommended that future research should consider how this measure may be individualised to a patient’s context.

No recommendations could be made for the domains of quality of life, patient rating of condition and psychology, primarily because there were no measures that had been validated for use in LET. For quality of life, the EQ5D narrowly missed interim selection and provoked mixed feelings from the patient focus groups. It was considered useful from a health economics perspective, for the calculation of quality adjusted life years.^30 31^ Of the three instruments considered for the patient rating of condition domain, the Global Rating of Change (GROC) was preferred by the patient focus groups and is also regarded as an appropriate anchor for responsiveness analysis of other outcome measures.^32 33^ The Single Assessment Numerical Evaluation^34^ was proposed as an alternative option with the advantage that it can be used pretreatment and posttreatment, rather than relying on symptom recall, like the GROC. For the psychology domain, patients indicated the Tampa Scale of Kinesiophobia^35^ was more representative of their condition than anxiety and depression scales. Future studies should investigate the psychometric properties of these instruments for the LET population.

### Strengths and Limitations

A strength of this study is that we included experts in LET (ie, patients and clinicians/researchers) from across the globe and followed a robust methodology that was published in advance.

There are several limitations that need to be considered in implementing the findings of this study. First, the COS-LET was developed on the basis of previously agreed on core domains for tendinopathy and are dependent on that work—any changes to those core domains will require revision of the COS-LET. Second, we were unable to recommend outcome measures for all of the core domains of tendinopathy—in which case we made interim recommendations. These interim recommendations should not be misconstrued as being part of the COS-LET, because they were made on the basis of opinions of participants without appropriate instrument validation. Third, we restricted the study to English outcome measures—using the COS-LET in non-English language situations requires validation. Fourth, we did not include a patient in our steering committee.

### Areas for future research

Future research is required to establish valid and feasible measures across all health-related tendinopathy domains in patients who have LET. We have identified some targets herein: PRTEE subscales/items, pain on gripping, GROC, EQ5D and Tampa Scale of Kinesiophobia.

## Conclusion

The PRTEE should be used in all future studies related to LET—especially for the disability domain. Time off work, pain-free grip strength and a Numerical Rating Scale measuring pain on gripping should also be used until future studies recommend alternative, more robust, measures of participation in life activities, physical function capacity and pain on activity/loading. A COS-LET Tool, containing these recommended measures, has been composed (see online supplemental file 4). Further work is required to (a) validate the interim measures for use in research involving persons with LET and (b) develop/validate suitable measures of the patient rating of condition, quality of life and psychological factors domains.

10.1136/bjsports-2021-105044.supp4 Supplementary data

**Key messages**

**What is already known?**

- Core outcome sets (COSs) are recommended for research and clinical practice to facilitate comparison and meta-analysis of results.
- COSs for tendinopathies should map to the established list of nine core health-related domains.
- Lateral elbow tendinopathy (LET) is a common clinical problem that has received considerable research attention.
- There is no agreed COS-LET—limiting meta-analysis.

**What are the findings?**

- The COS-LET consists of the Patient Rated Tennis Elbow Evaluation—it should be used to capture the disability domain in clinical settings and in all research of this condition.
- The Patient Rated Tennis Elbow Evaluation and its subscales offer insights into the domains of pain and function in addition to disability.
- When measuring participation, physical function capacity and pain on loading, we recommend as interim measures, respectively, time off work, pain-free grip strength and a numerical rating scale for pain on gripping.
- Further work is required to validate many of the measures used in clinical practice.

How might it impact on clinical practice in the future?

- Systematic use of the COS-LET will allow for meta-analysis of research studies and comparison of results between different clinical practices during service evaluation.
- Meta-analysis and network meta-analysis across multiple research trials that apply the COS-LET will increase understanding of treatment effectiveness for people with LET.

## Data Availability

All data relevant to the study are included in the article or uploaded as supplementary information. Not applicable.
