# Supplementary material for: Development of a core outcome set for lateral elbow tendinopathy (COS-LET) using best available evidence and an international consensus process
Source: Br J Sports Med. 2022 Feb 8;56(12):657–66. doi: 10.1136/bjsports-2021-105044 (PMC9163713; doi:10.1136/bjsports-2021-105044)

Delphi Process for Core Outcome Set for Lateral Elbow Tendinopathy (COS-LET): Round 1 Survey Data

Context:

1. There is a high level of heterogeneity in outcome measures used in trials of lateral elbow tendinopathy (LET), which makes evidence synthesis across studies difficult.
2. Previous work in the field of tendinopathy has established through a consensus exercise nine core health-related domains that should be measured in tendinopathy research.
3. The aim of this study is to develop a Core Outcome Set for Lateral Elbow Tendinopathy (COS-LET) mapping to these core domains.

Methods:

The development of the COS-LET is being developed as per the following process:

1. Systematic review of studies investigating LET has revealed a comprehensive list of all instruments that have previously been used to quantify treatment effect or outcome.
2. These instruments were matched to the list of nine core tendinopathy outcome domains by a Steering Committee of clinicians and researchers with a specialist interest in LET resulting in a set of candidate instruments.
3. A 3-stage international consensus process involving experienced clinicians, researchers and patients will be conducted to determine agreement on what should be the COS-LET.
4. We, including you, have completed the 1st stage, which was to respond to a survey.
    - The committee has now collated your responses – reported herein – and then reviewed the psychometric/clinimetric literature to find and rate the available data on the measures you the responders considered should be in a COS-LET.
    - The second stage is where we are at now – the following survey will seek out your responses to a series of questions about including or not measures in a COS-LET.

Results:

The results of the first survey of healthcare professionals and patients are shown herein in Table 1 and 2, and Figures 1-10. **Table 1** shows the characteristics of the healthcare professionals and patients responding to the survey. **Table 2** is a summary of the results with data representing % agreement for each instrument. **Figures 1-10** illustrates the absolute number and % agreement, disagreement and unsure for each instrument.

In summary, there were four instruments that were above the 70% agreement threshold for inclusion in the COS-LET based on responses from healthcare professionals. Of these instruments, three were also above the 70% agreement threshold based on responses from patients:

- VAS pain on gripping (in the Pain on Activity or Loading Domain)
- Patient-Rated Tennis Elbow Evaluation (in the Disability Domain)
- Quick DASH (in the Disability Domain)

There were a number of instruments that were above the 70% disagreement threshold for inclusion in the COS-LET based on responses from both healthcare professionals and patients: 2 in the Pain on Activity or Loading Domain; 9 in the Disability Domain; 1 in the Quality of Life Domain; and 12 that were not mapped to any of the 9 core health-related domains for tendinopathy. These instruments were subsequently excluded.

There were eight instruments that were not previously included that were listed as important or critical for inclusion. These are highlighted in yellow in Table 1.

Table 1: Participant Characteristics (n (%) unless otherwise stated) of those who completed the full survey and provided these details (39 participants commenced, but did not complete).

Characteristics	Healthcare Professionals (N=37)	Patients (N=7)
Sex: Male	25 (67.6)	2 (28.6)
Age: median (IQR; min-max) years	51 (43-57; 34-68)	48 (47.5-54.5; 26-59)
Role:		
Clinician	2 (5.4)	
Researcher	5 (13.5)	
Clinician Researcher	30 (81.1)	
Not a Clinician or Researcher		7 (100)
Highest academic qualification:		
PhD	21 (56.8)	
Master	6 (16.2)	2 (28.6)
Doctor of Medicine	6 (16.2)	
Bachelor	3 (8.1)	3 (42.9)
Undergraduate Diploma/Certificate		1 (14.3)
Not specified	1 (2.7)	
No university qualification		1 (14.3)
Profession:		
Physiotherapist	16 (43.2)	
Orthopaedic surgeon	14 (37.8)	
Sports & Exercise Medicine Physician	3 (8.1)	
Not specified	3 (8.1)	
Rheumatologist	1 (2.7)	
Patient		7 (100%)
Lateral elbow tendinopathy:		
Current history	1 (2.7)	5 (71.4)
Past history	10 (27.0)	4 (57.1)
Country where work:		
Australia	11 (29.7)	2 (28.6)
United Kingdom	10 (27.0)	5 (71.4)
USA	3 (8.1)	
Canada and Norway each:	2 (5.4)	
Belgium, Finland, Greece, Israel, Italy, Netherlands, Spain, Sweden, and Turkey each:	1 (2.7)	

Table 1: Summary of Round 1 Results: data are % responses, with green representing >70% agree, red >70% disagree, and amber neither green or red.

	Health Care Professionals (N = 39^)				Patients (N = 7)		
	**In COS-LET?**	**Truth (a)**	**Feasibility**		**In COS-LET?**	**Truth (a)**	**Feasibility**
**Patient Rating of Condition** Domain							
Global Perceived Effect score	56.41	71.79	92.31		71.43	85.71	85.71
Global Rating of Change	64.1	74.36	87.18		57.14	85.71	85.71
Patient Satisfaction Scale	51.28	64.1	89.74		71.43	85.71	85.71
Roles & Maudsley Score	Proposed in Survey 1 comments						
**Participation in Live Activities** Domain							
Return to sport	38.46	64.1	82.05		71.43	71.42	85.71
Time off work	53.85	79.49	76.92		57.14	57.14	85.71
Total Elbow Scoring System	30.77	35.9	53.85		57.14	57.14	71.43
OSTRC Question 1	Proposed in Survey 1 comments						
**Pain on Activity or Loading** Domain							
Tennis Elbow Functional Scale*	28.21	61.54	56.41		85.71	85.71	85.71
Thomsen Test	35.9	71.79	71.79		71.43	71.43	85.71
VAS chair pick-up	23.08	51.28	58.97		28.57	85.71	57.14
VAS pain during activity	79.49	89.74	87.18		57.14	71.43	85.71
VAS pain during elbow movement	17.95	33.33	69.23		28.57	42.86	85.71
VAS pain on gripping	71.79	94.87	89.74		71.43	71.43	100
VAS pain at work	38.46	58.97	74.36		42.86	42.86	57.14
Pain-Free Functional Index	17.95	51.28	58.97		42.86	42.86	85.71
Patient-Rated Tennis Elbow Evaluation*	61.54	79.49	71.79		100	100	100
**Function** Domain							
Patient Specific Functional Scale	28.95	63.16	52.63		71.43	85.71	85.71
Upper Extremity Functional Scale	44.74	68.42	68.42		57.14	57.14	100
VAS function	44.74	63.16	76.32		28.57	42.86	57.14
Patient-Rated Tennis Elbow Evaluation*	Proposed in Survey 1 comments						
**Psychological Factors** Domain							
Hospital Anxiety and Depression Scale	36.84	52.63	50		28.57	28.57	85.71
Tampa Scale of Kinesophobia	39.47	50	55.26		71.43	85.71	71.43
State-Trait Anxiety Inventory	Proposed in Survey 1 comments						
Nottingham Health Profile	Proposed in Survey 1 comments						
**Physical Function Capacity** Domain							
Grip strength (maximum)*	47.37	81.58	68.42		85.71	85.71	100
Pain free grip strength*	65.79	78.95	71.05		85.71	85.71	100
Elbow ROM	10.53	31.58	65.79		42.86	28.57	100

**Disability** Domain						
Andrews-Carson Score	0	7.89	26.32	14.29	14.29	42.86
American Shoulder & Elbow Score	10.53	44.74	34.21	42.86	42.86	71.43
Broberg & Morrey Rating System	2.63	21.05	26.32	28.57	42.86	71.43
Disabilities of the Arm Shoulder and Hand*	39.47	68.42	44.74	57.14	57.14	71.43
HAND10	7.89	44.74	60.53	57.14	57.14	85.71
Japanese Orthopaedic Association Elbow Score	10.53	44.74	42.11	0	0	57.14
Laitinen Questionnaire	13.16	36.84	39.47	14.29	14.29	57.14
Liverpool Elbow Score	2.63	34.21	34.21	28.57	42.86	42.86
Mayo Elbow Performance Score	7.89	31.58	39.47	0	0	28.57
Nirschl Tennis Elbow Score	18.42	57.89	55.26	57.14	57.14	57.14
Nottingham Health Profile	2.63	15.79	31.58	0	0	0
Oxford Elbow Score*	23.68	52.63	55.26	57.14	71.43	85.71
Patient-Rated Wrist Evaluation Questionnaire	13.16	44.74	50.00	57.14	57.14	85.71
Patient-Rated Tennis Elbow Evaluation*	73.68	92.11	86.84	85.71	85.71	85.71
Quick DASH*	71.05	76.32	81.58	100	100	100
Total Elbow Scoring System	10.53	36.84	52.63	28.57	28.57	57.14
Roles & Maudsley Score	5.26	28.95	57.89	14.29	28.57	57.14
**Quality of Life** Domain						
EuroQoL (EQ5D)	51.35	59.46	67.57	57.14	57.14	85.71
Short Form Survey (SF-36)	16.22	62.16	24.32	14.29	71.43	28.57
SF-12 Health Survey (SF-12)	40.54	67.57	67.57	42.86	71.43	57.14
Nottingham Health Profile	Proposed in Survey 1 comments					
World Health Organization Quality of Life Instruments (WHOQOL-BREF)	Proposed in Survey 1 comments					
Short Musculoskeletal Function Assessment	Proposed in Survey 1 comments					
**Pain Over a Specified Timeframe** Domain						
VAS night pain	37.84	70.27	86.49	28.57	57.14	85.71
VAS pain defined time period	67.57	81.08	78.38	28.57	42.86	71.43
VAS pain at rest	56.76	67.57	83.78	14.29	28.57	71.43
Tennis Elbow Functional Scale*	21.62	59.46	56.76	85.71	85.71	85.71
**Others**						
Analgesic use	43.24			14.29		
Canadian Occupational Performance Measure	5.41			14.29		
Cold Pain Threshold	8.11			14.29		
Electromyography	0			28.57		
Gothenburg Quality of Life Instrument	0			0		
MRI evaluation	8.11			14.29		

Orthopaedic Research Institute-Tennis Elbow Testing System	2.7			14.29		
Placzek Score	10.81			28.57		
Pressure Pain Threshold	18.92			14.29		
Ultrasound Appearance	10.81			14.29		
University of Peloponnese Pain, Functionality and Quality of Life Questionnaire	2.7			14.29		
VAS pain on palpation	10.81			28.57		
VAS pain overall	24.32			28.57		
Work Limitations Questionnaire	5.41			57.14		

^ 39 responders completed the first 3 domains, 38 continued on to the next 4 domains and 37 completed it all

**indicates the measures that had clinimetric data/study(ies) with a score of at least 40%.*

Figure 1: Participant response for **Patient Rating of Condition** Domain

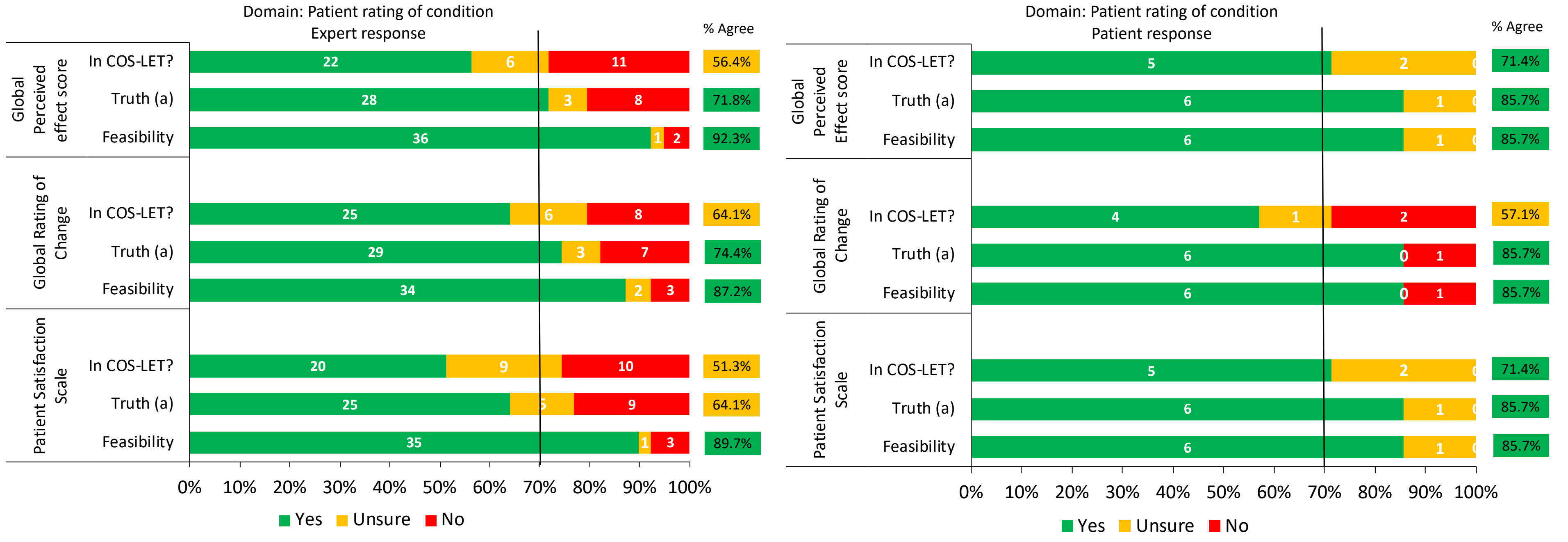

Figure 2: Participant response for **Participation in Life Activities** Domain

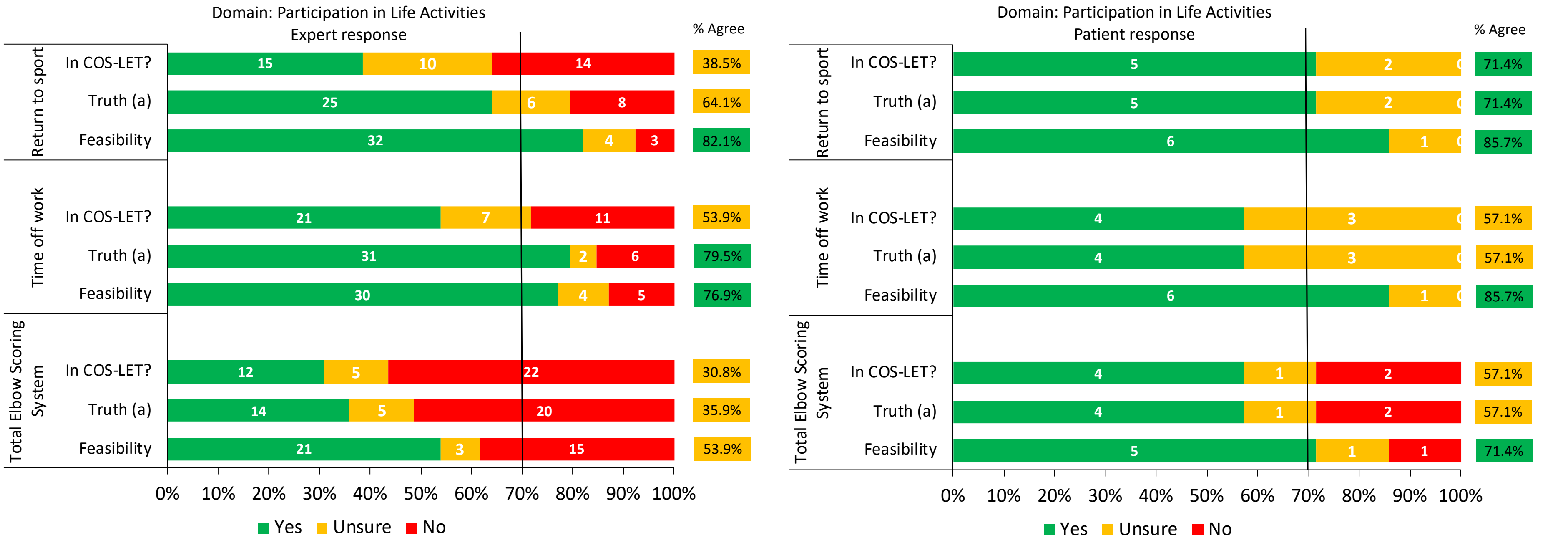

Figure 3: Participant response for **Pain on Activity or Loading** Domain

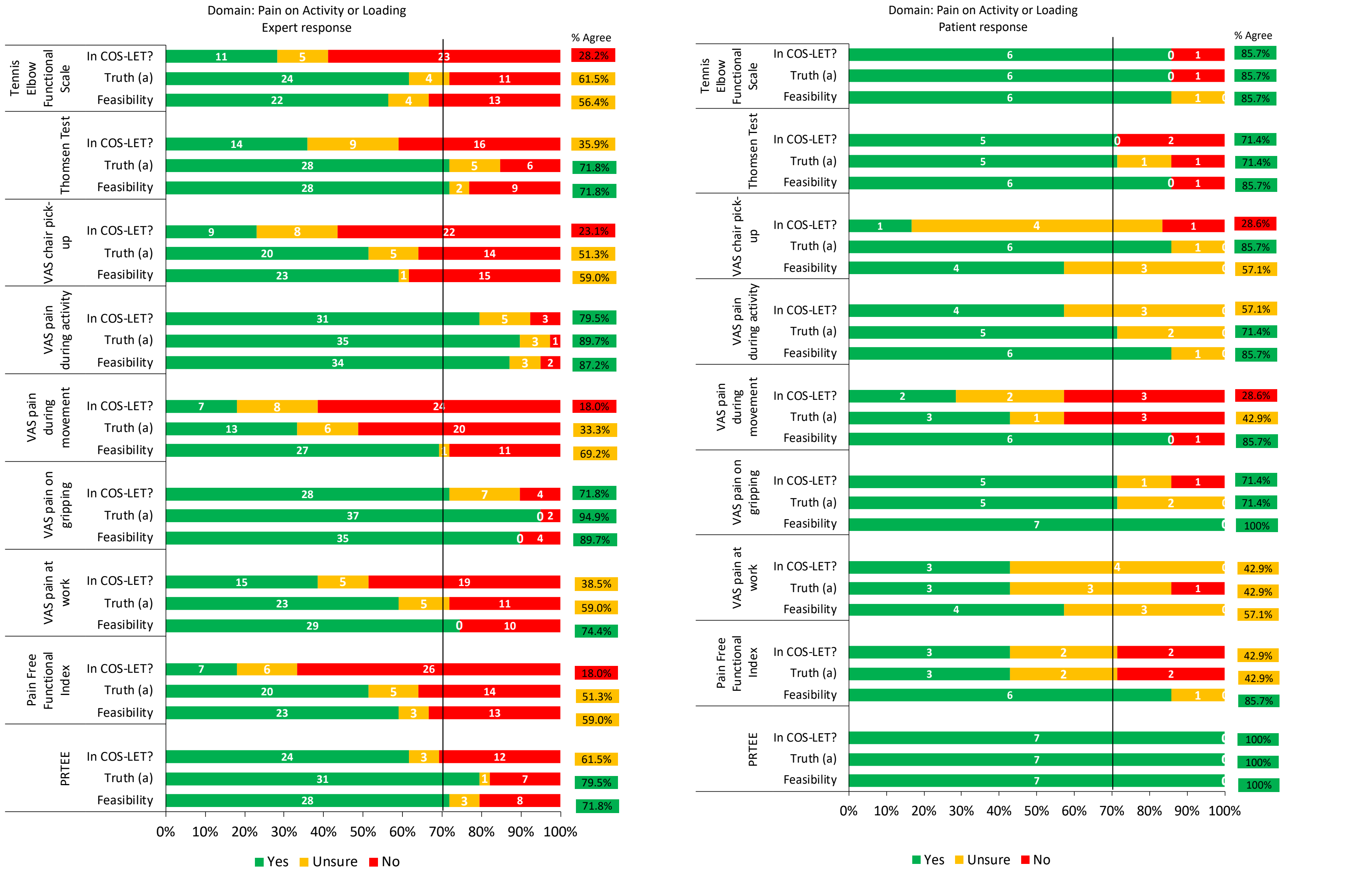

Figure 4: Participant response for **Function** Domain

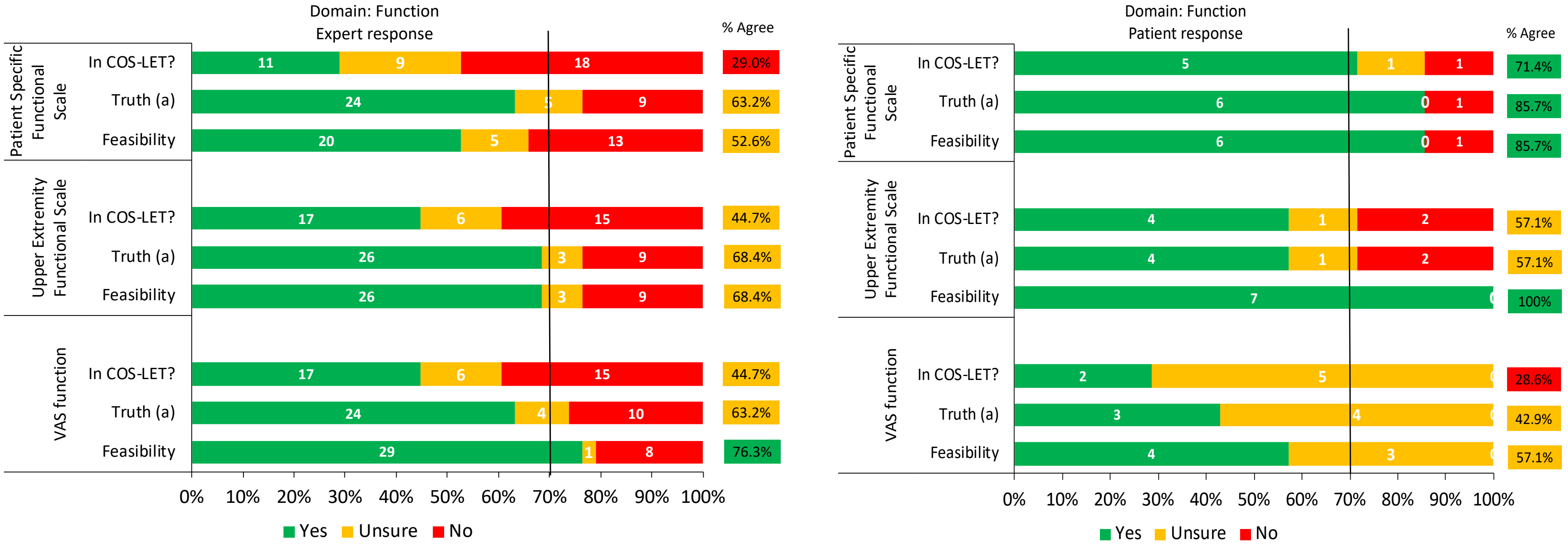

Figure 5: Participant response for **Psychological Factors** Domain

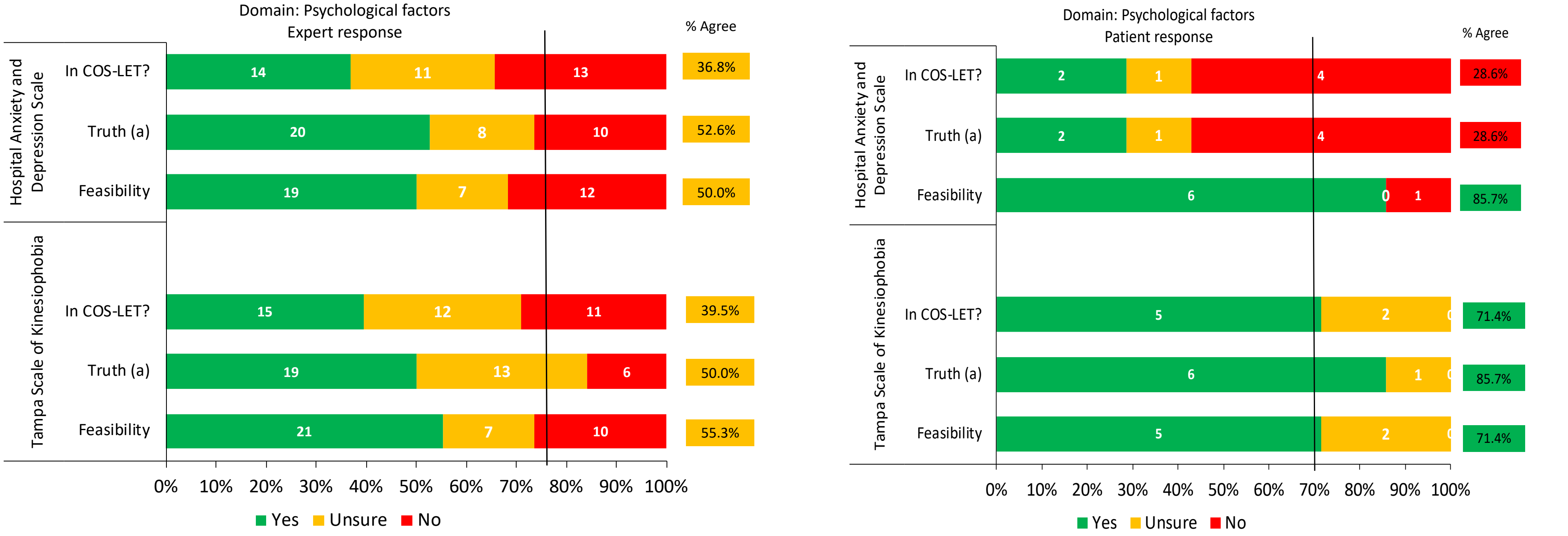

Figure 6: Participant response for **Physical Function Capacity** Domain

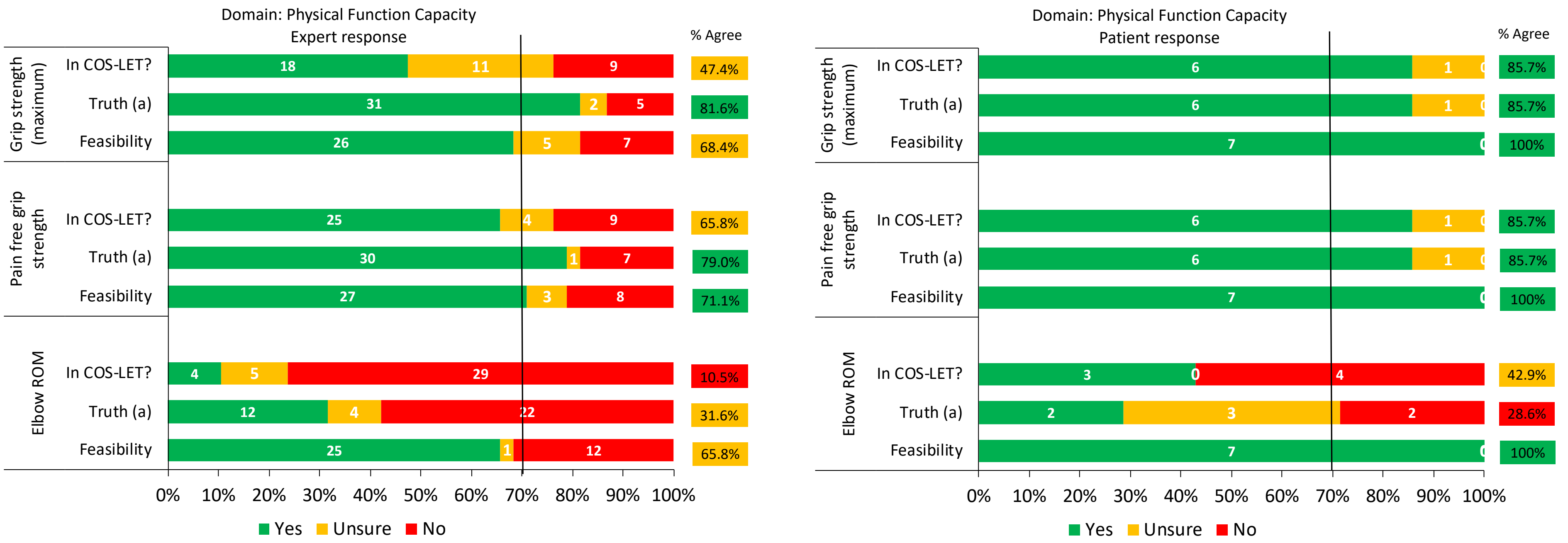

Figure 7: Participant response for **Disability** Domain

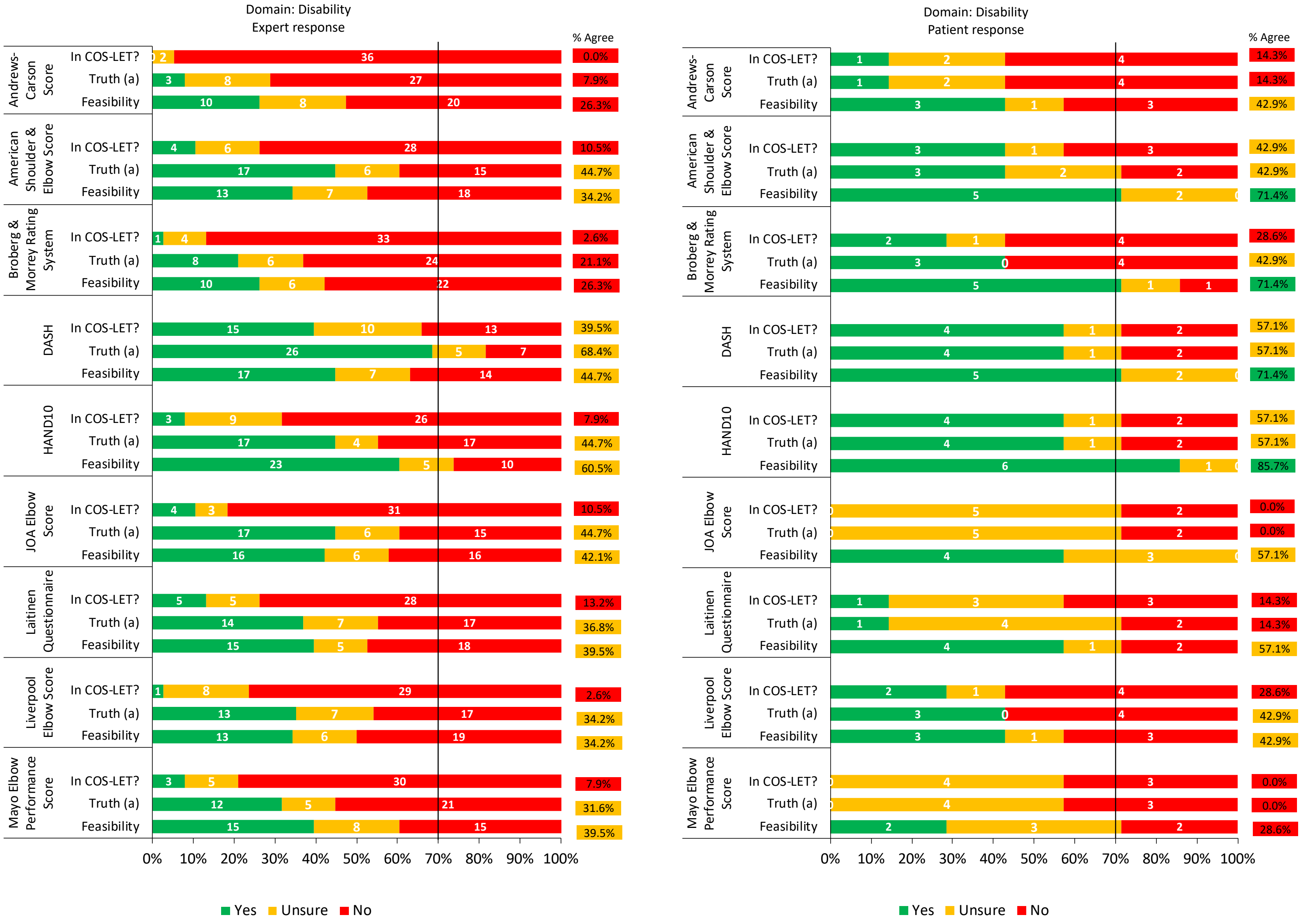

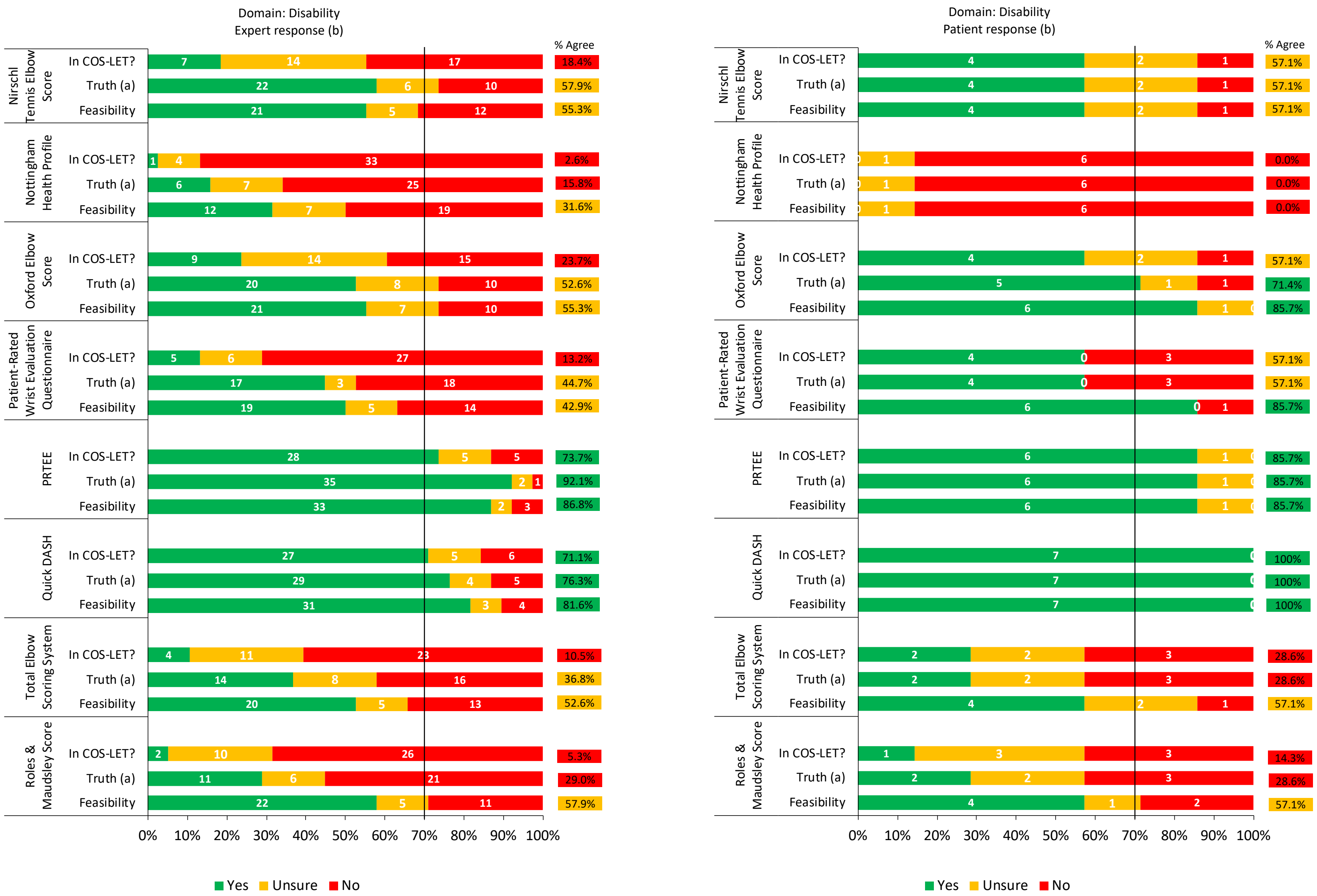
Domain: Disability
Expert response (b)
% Agree
Nirschl Tennis Elbow Score
Nottingham Health Profile
Oxford Elbow Score
Patient-Rated Wrist Evaluation Questionnaire
PRTEE
Quick DASH
Total Elbow Scoring System
Roles & Maudsley Score
In COS-LET?
Truth (a)
Feasibility
Yes
Unsure
No
Domain: Disability
Patient response (b)

Figure 8: Participant response for **Quality of Life** Domain

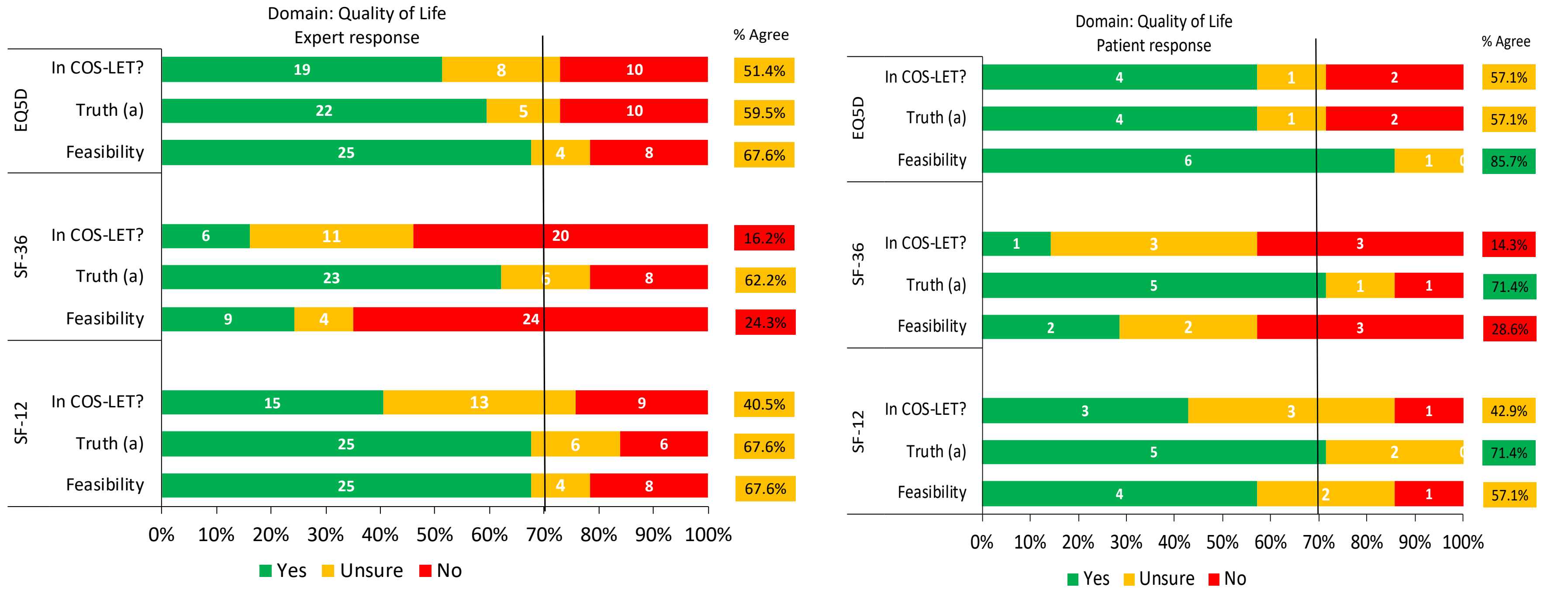

Figure 9: Participant response for **Pain Over a Specified Time** Domain

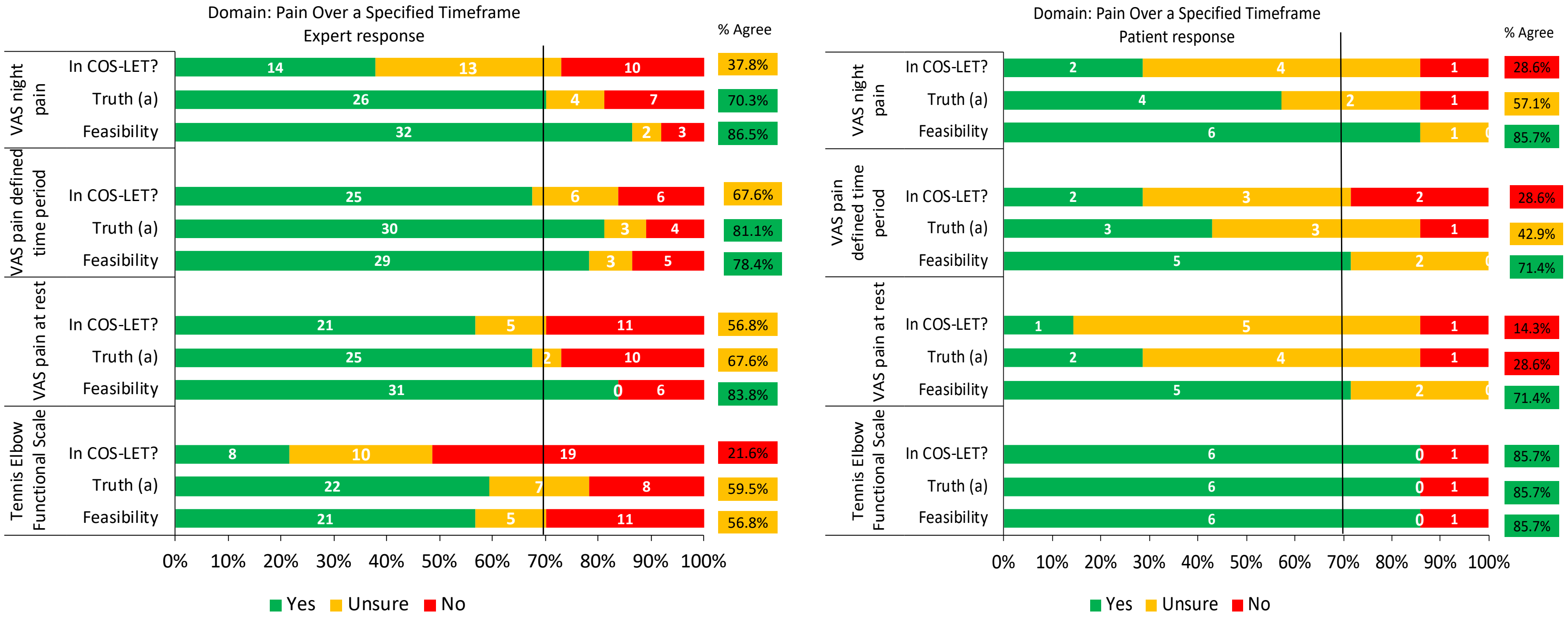

Figure 10: Other outcome measures

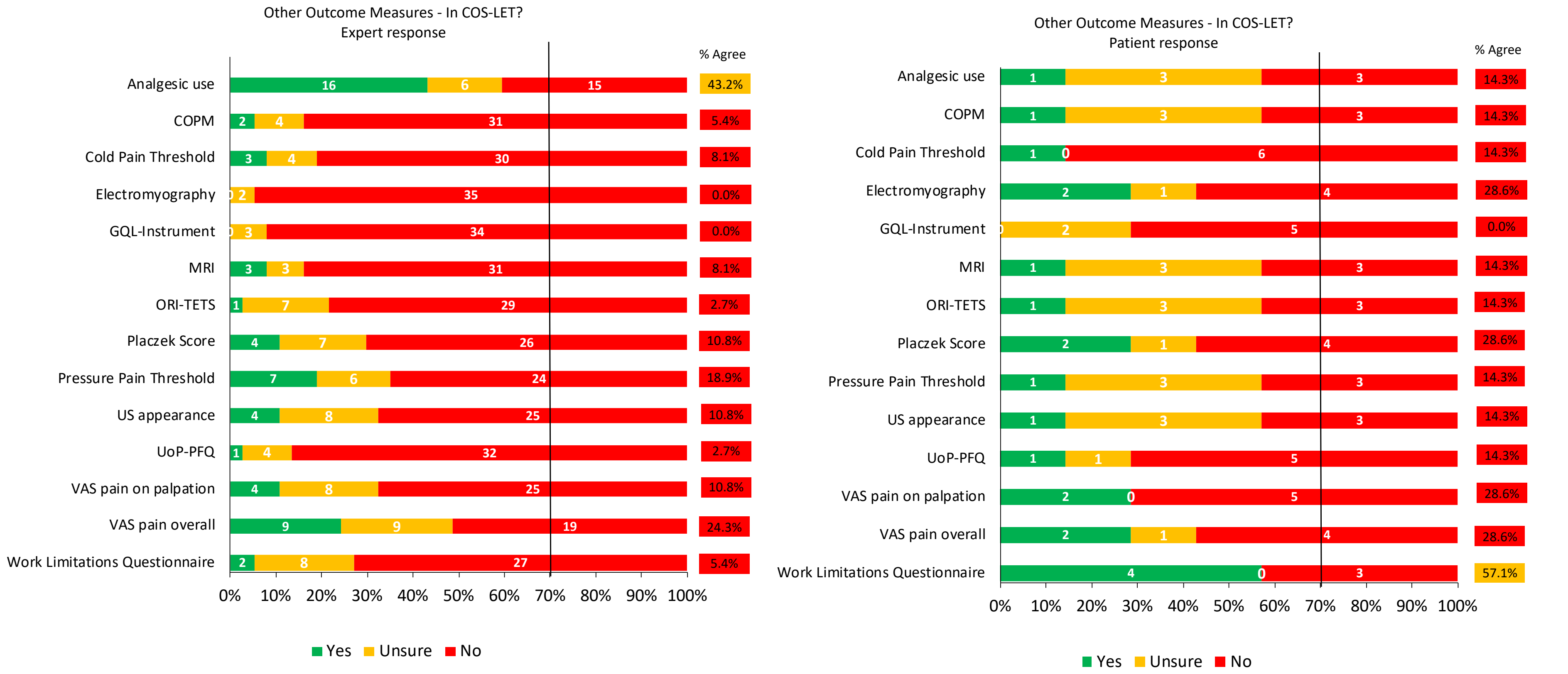

Supplement: Supplementary data [file bjsports-2021-105044supp001.pdf]
