# Supplementary material for: Development of a core outcome set for lateral elbow tendinopathy (COS-LET) using best available evidence and an international consensus process
Source: Br J Sports Med. 2022 Feb 8;56(12):657–66. doi: 10.1136/bjsports-2021-105044 (PMC9163713; doi:10.1136/bjsports-2021-105044)
Supplement: Supplementary data [file bjsports-2021-105044supp002.pdf]

Default Report

COS-LET_Steering_Committee_OMERACT-filter2
January 10, 2021 5:43 PM MST

Q49 - Please leave your details here in case we need to follow up.

Last name	First name
val	jones
Phadnis	Joideep
Watts	Adam
Evans	Jon
Bisset	Leanne
Vicenzino	Bill
Vuvan	Viana
Bateman	Marcus

truth - The PRTEE meets the truth OMERACT filter requirements for the outcome measure representing the Disability Domain in the core outcome set for lateral elbow tendinopathy.

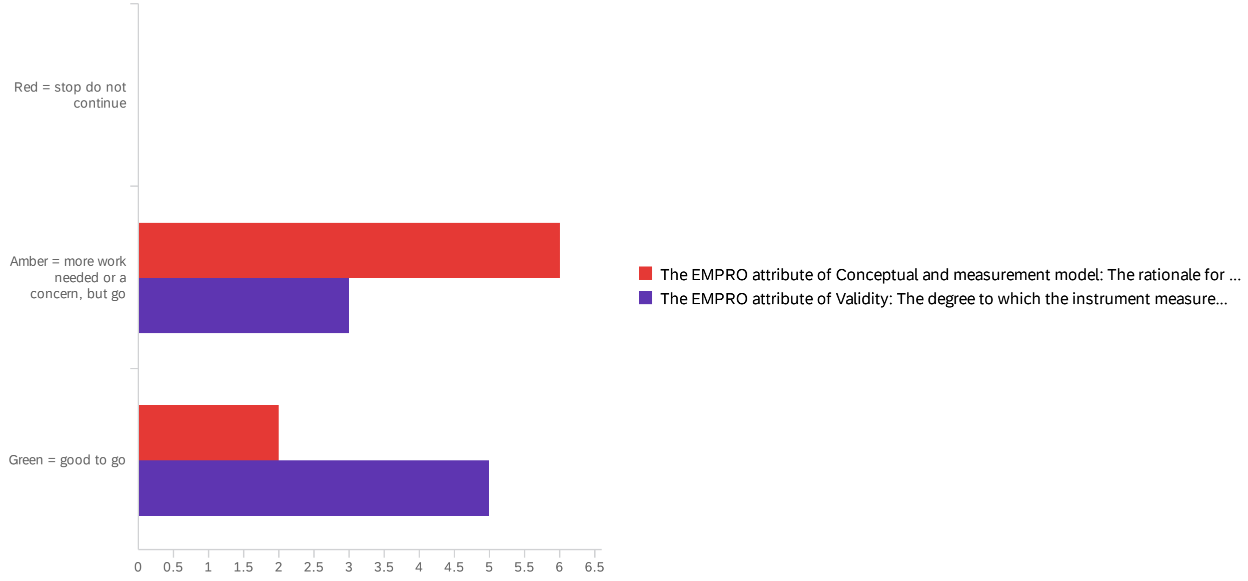

#	Field	Minimum	Maximum	Mean	Std Deviation	Variance	Count
1	The EMPRO attribute of Conceptual and measurement model: The rationale for and description of the concept and the populations that a measure is intended to assess and the relationship between these concepts: score of 42.86%	2.00	3.00	2.25	0.43	0.19	8
2	The EMPRO attribute of Validity: The degree to which the instrument measures what it purports to measure: score of 53.33%	2.00	3.00	2.63	0.48	0.23	8

#	Field	Red = stop do not continue		Amber = more work needed or a concern, but go		Green = good to go		Total
1	The EMPRO attribute of Conceptual and measurement model: The rationale for and description of the concept and the populations that a measure is intended to assess and the relationship between these concepts: score of 42.86%	0.00%	0	75.00%	6	25.00%	2	8
2	The EMPRO attribute of Validity: The degree to which the instrument measures what it purports to measure: score of 53.33%	0.00%	0	37.50%	3	62.50%	5	8

Showing rows 1 - 2 of 2

discrim - PRTEE meets the discrimination OMERACT filter requirements for the outcome measure representing the Disability Domain in the core outcome set for lateral elbow tendinopathy.

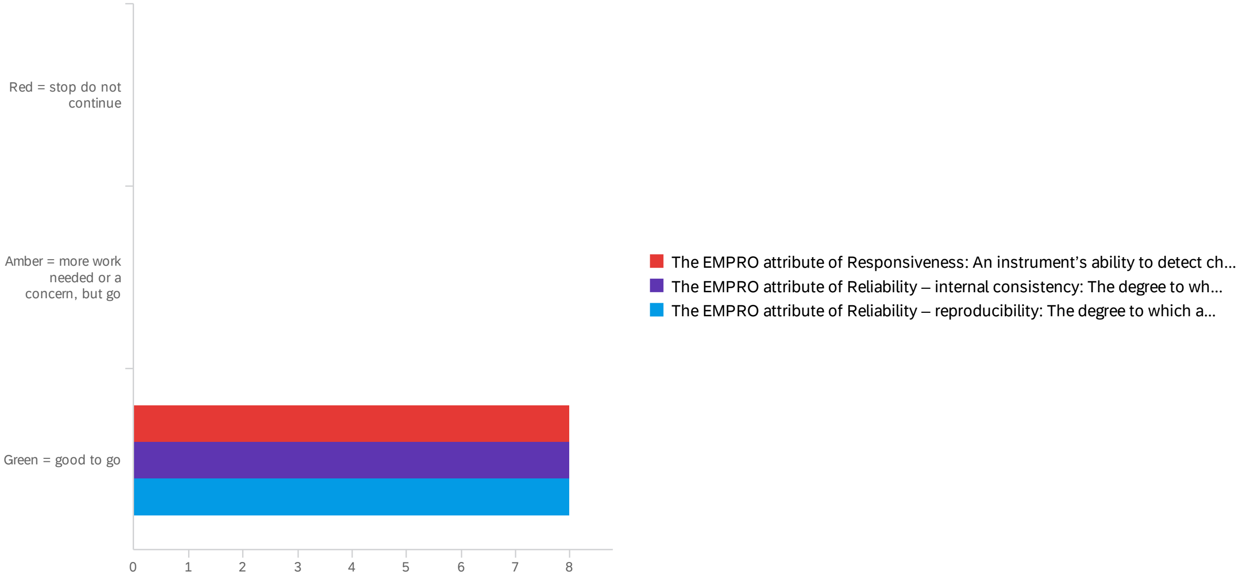

#	Field	Minimum	Maximum	Mean	Std Deviation	Variance	Count
1	The EMPRO attribute of Responsiveness: An instrument's ability to detect change over time: score of 77.78%	3.00	3.00	3.00	0.00	0.00	8
2	The EMPRO attribute of Reliability – internal consistency: The degree to which an instrument is free from random error: score of 66.67%	3.00	3.00	3.00	0.00	0.00	8
3	The EMPRO attribute of Reliability – reproducibility: The degree to which an instrument is free from random error: score of 66.67%	3.00	3.00	3.00	0.00	0.00	8

#	Field	Red = stop do not continue	Amber = more work needed or a concern, but go	Green = good to go	Total
1	The EMPRO attribute of Responsiveness: An instrument's ability to detect change over time: score of 77.78%	0.00% 0	0.00% 0	100.00% 8	8
2	The EMPRO attribute of Reliability – internal consistency: The degree to which an instrument is free from random error: score of 66.67%	0.00% 0	0.00% 0	100.00% 8	8

#	Field	Red = stop do not continue		Amber = more work needed or a concern, but go		Green = good to go		Total
3	The EMPRO attribute of Reliability – reproducibility: The degree to which an instrument is free from random error: score of 66.67%	0.00%	0	0.00%	0	100.00%	8	8

Showing rows 1 - 3 of 3

overall - Considering your responses to the truth and discrimination OMERACT filter above, does PRTEE meets the requirements for the outcome measure representing the Disability Domain in the core outcome set for lateral elbow tendinopathy.

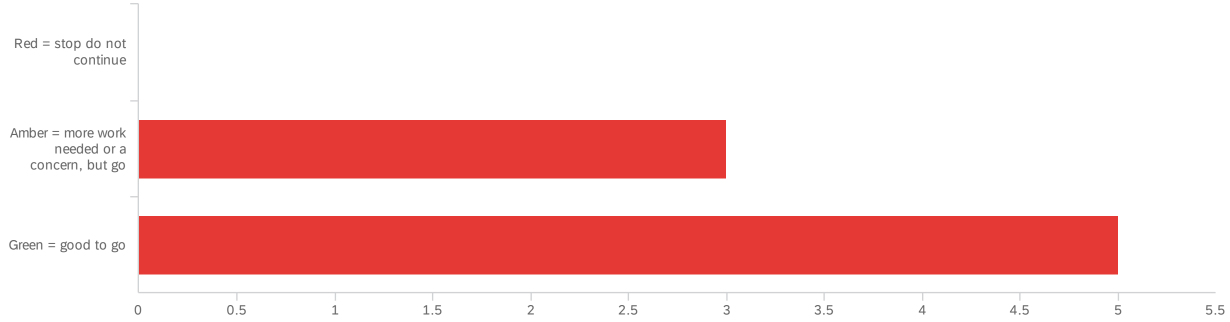

#	Field	Minimum	Maximum	Mean	Std Deviation	Variance	Count
1	The overal EMPRO Score (a composite of the above attribute scores) is: 57.02%	2.00	3.00	2.63	0.48	0.23	8

#	Field	Choice Count	
1	Red = stop do not continue	0.00%	0
2	Amber = more work needed or a concern, but go	37.50%	3
3	Green = good to go	62.50%	5
			8

Showing rows 1 - 4 of 4

Q12 - The qDASH meets the truth OMERACT filter requirements for the outcome measure representing the Disability Domain in the core outcome set for lateral elbow tendinopathy.

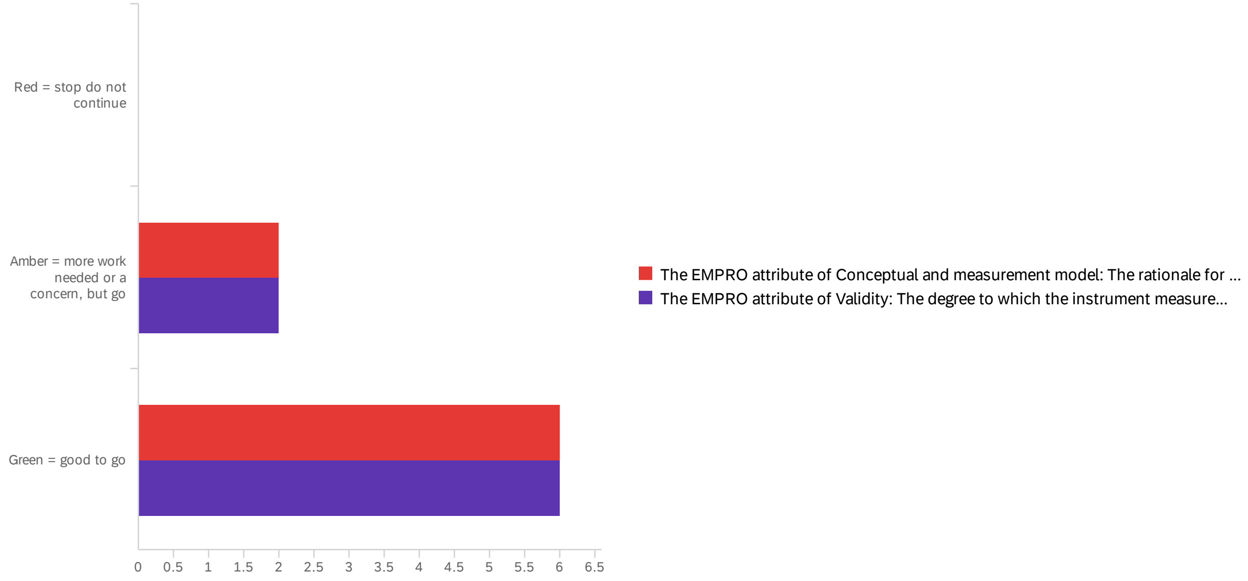

#	Field	Minimum	Maximum	Mean	Std Deviation	Variance	Count
1	The EMPRO attribute of Conceptual and measurement model: The rationale for and description of the concept and the populations that a measure is intended to assess and the relationship between these concepts: score of 76.19%	2.00	3.00	2.75	0.43	0.19	8
2	The EMPRO attribute of Validity: The degree to which the instrument measures what it purports to measure: score of 72.22%	2.00	3.00	2.75	0.43	0.19	8

#	Field	Red = stop do not continue		Amber = more work needed or a concern, but go		Green = good to go		Total
1	The EMPRO attribute of Conceptual and measurement model: The rationale for and description of the concept and the populations that a measure is intended to assess and the relationship between these concepts: score of 76.19%	0.00%	0	25.00%	2	75.00%	6	8
2	The EMPRO attribute of Validity: The degree to which the instrument measures what it purports to measure: score of 72.22%	0.00%	0	25.00%	2	75.00%	6	8

Showing rows 1 - 2 of 2

Q13 - qDASH meets the discrimination OMERACT filter requirements for the outcome measure representing the Disability Domain in the core outcome set for lateral elbow tendinopathy.

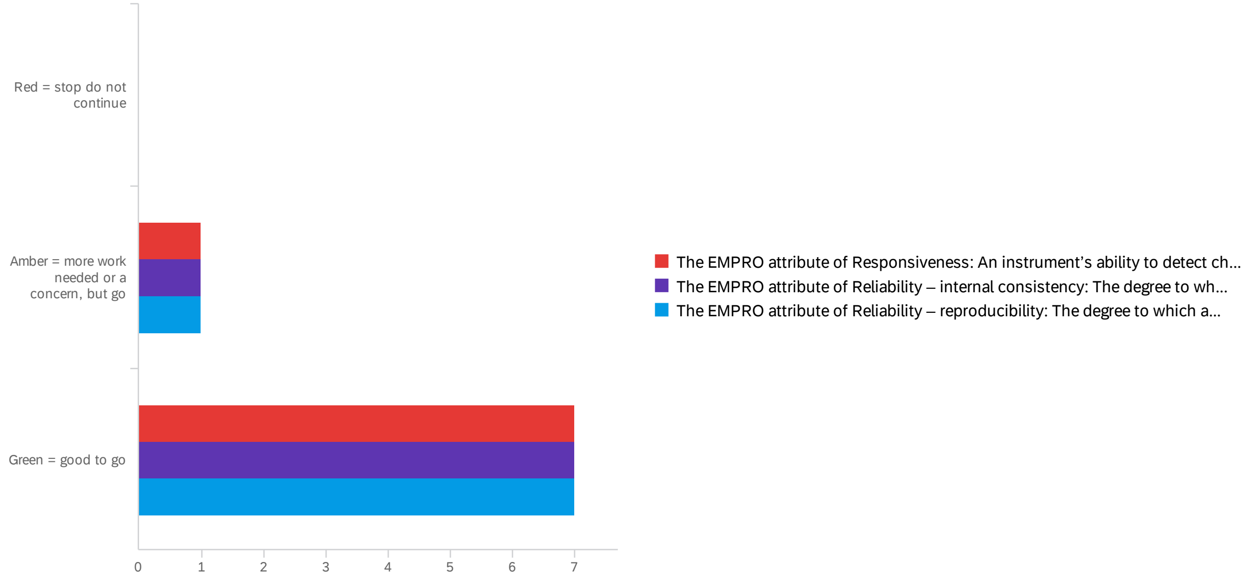

#	Field	Minimum	Maximum	Mean	Std Deviation	Variance	Count
1	The EMPRO attribute of Responsiveness: An instrument's ability to detect change over time: score of 77.78%	2.00	3.00	2.88	0.33	0.11	8
2	The EMPRO attribute of Reliability – internal consistency: The degree to which an instrument is free from random error: score of 66.67%	2.00	3.00	2.88	0.33	0.11	8
3	The EMPRO attribute of Reliability – reproducibility: The degree to which an instrument is free from random error: score of 66.67%	2.00	3.00	2.88	0.33	0.11	8

#	Field	Red = stop do not continue		Amber = more work needed or a concern, but go		Green = good to go		Total
1	The EMPRO attribute of Responsiveness: An instrument's ability to detect change over time: score of 77.78%	0.00%	0	12.50%	1	87.50%	7	8
2	The EMPRO attribute of Reliability – internal consistency: The degree to which an instrument is free from random error: score of 66.67%	0.00%	0	12.50%	1	87.50%	7	8

#	Field	Red = stop do not continue		Amber = more work needed or a concern, but go		Green = good to go		Total
3	The EMPRO attribute of Reliability – reproducibility: The degree to which an instrument is free from random error: score of 66.67%	0.00%	0	12.50%	1	87.50%	7	8

Showing rows 1 - 3 of 3

Q14 - Considering your responses to the truth and discrimination OMERACT filter above, does qDASH meets the requirements for the outcome measure representing the Disability Domain in the core outcome set for lateral elbow tendinopathy.

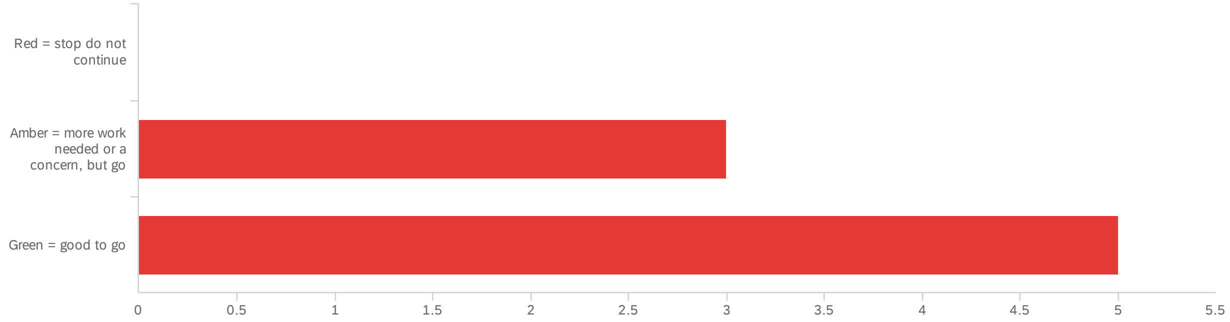

#	Field	Minimum	Maximum	Mean	Std Deviation	Variance	Count
1	The overal EMPRO Score (a composite of the above attribute scores) is: 57.02%	2.00	3.00	2.63	0.48	0.23	8

#	Field	Choice Count	
1	Red = stop do not continue	0.00%	0
2	Amber = more work needed or a concern, but go	37.50%	3
3	Green = good to go	62.50%	5
			8

Showing rows 1 - 4 of 4

Q16 - The DASH meets the truth OMERACT filter requirements for the outcome measure representing the Disability Domain in the core outcome set for lateral elbow tendinopathy.

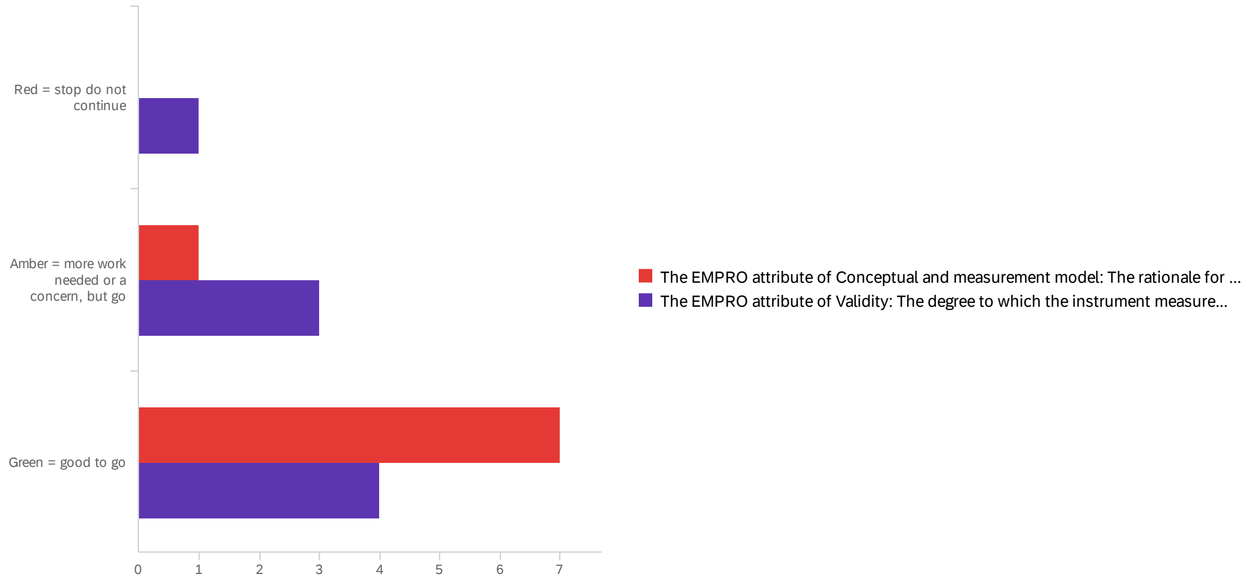

#	Field	Minimum	Maximum	Mean	Std Deviation	Variance	Count
1	The EMPRO attribute of Conceptual and measurement model: The rationale for and description of the concept and the populations that a measure is intended to assess and the relationship between these concepts: score of 76.19%	2.00	3.00	2.88	0.33	0.11	8
2	The EMPRO attribute of Validity: The degree to which the instrument measures what it purports to measure: score of 50%	1.00	3.00	2.38	0.70	0.48	8

#	Field	Red = stop do not continue		Amber = more work needed or a concern, but go		Green = good to go		Total
1	The EMPRO attribute of Conceptual and measurement model: The rationale for and description of the concept and the populations that a measure is intended to assess and the relationship between these concepts: score of 76.19%	0.00%	0	12.50%	1	87.50%	7	8
2	The EMPRO attribute of Validity: The degree to which the instrument measures what it purports to measure: score of 50%	12.50%	1	37.50%	3	50.00%	4	8

Showing rows 1 - 2 of 2

Q17 - DASH meets the discrimination OMERACT filter requirements for the outcome measure representing the Disability Domain in the core outcome set for lateral elbow tendinopathy.

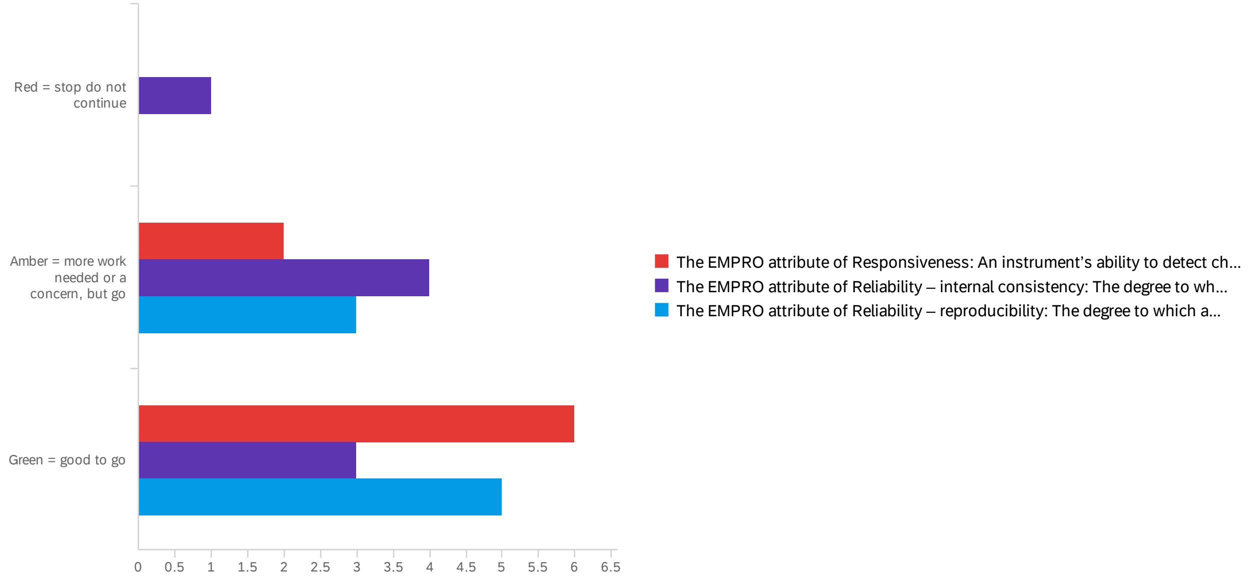

#	Field	Minimum	Maximum	Mean	Std Deviation	Variance	Count
1	The EMPRO attribute of Responsiveness: An instrument's ability to detect change over time: score of 66.67%	2.00	3.00	2.75	0.43	0.19	8
2	The EMPRO attribute of Reliability – internal consistency: The degree to which an instrument is free from random error: score of 50%	1.00	3.00	2.25	0.66	0.44	8
3	The EMPRO attribute of Reliability – reproducibility: The degree to which an instrument is free from random error: score of 75%	2.00	3.00	2.63	0.48	0.23	8

#	Field	Red = stop do not continue	Amber = more work needed or a concern, but go	Green = good to go	Total
1	The EMPRO attribute of Responsiveness: An instrument's ability to detect change over time: score of 66.67%	0.00% 0	25.00% 2	75.00% 6	8
2	The EMPRO attribute of Reliability – internal consistency: The degree to which an instrument is free from random error: score of 50%	12.50% 1	50.00% 4	37.50% 3	8

#	Field	Red = stop do not continue		Amber = more work needed or a concern, but go		Green = good to go		Total
3	The EMPRO attribute of Reliability – reproducibility: The degree to which an instrument is free from random error: score of 75%	0.00%	0	37.50%	3	62.50%	5	8

Showing rows 1 - 3 of 3

Q18 - Considering your responses to the truth and discrimination OMERACT filter above, does DASH meets the requirements for the outcome measure representing the Disability Domain in the core outcome set for lateral elbow tendinopathy.

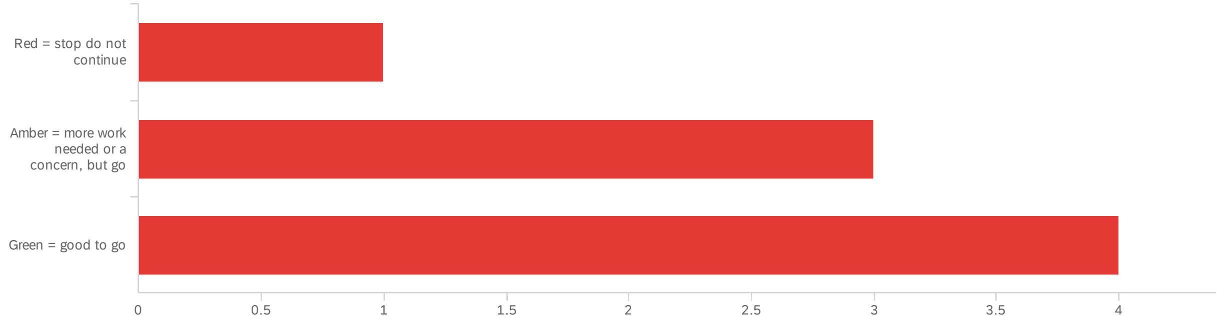

#	Field	Minimum	Maximum	Mean	Std Deviation	Variance	Count
1	The overal EMPRO Score (a composite of the above attribute scores) is: 66.9%	1.00	3.00	2.38	0.70	0.48	8

#	Field		Choice Count
1	Red = stop do not continue	12.50%	1
2	Amber = more work needed or a concern, but go	37.50%	3
3	Green = good to go	50.00%	4
			8

Showing rows 1 - 4 of 4

Q20 - The OES meets the truth OMERACT filter requirements for the outcome measure representing the Disability Domain in the core outcome set for lateral elbow tendinopathy.

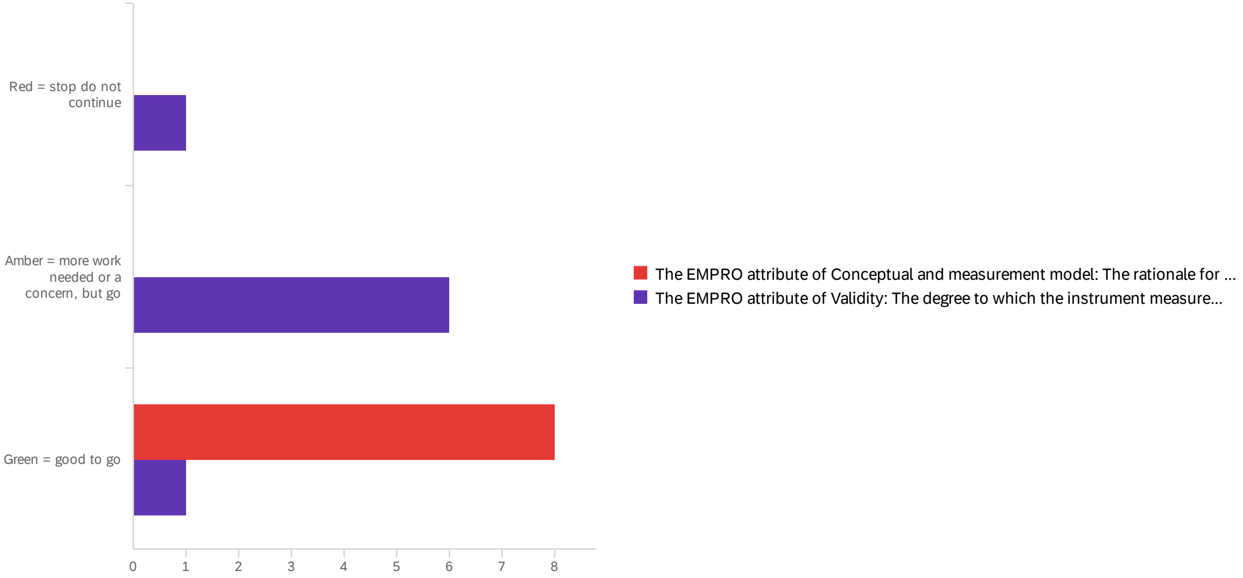

#	Field	Minimum	Maximum	Mean	Std Deviation	Variance	Count
1	The EMPRO attribute of Conceptual and measurement model: The rationale for and description of the concept and the populations that a measure is intended to assess and the relationship between these concepts: score of 85.7%	3.00	3.00	3.00	0.00	0.00	8
2	The EMPRO attribute of Validity: The degree to which the instrument measures what it purports to measure: score of 44.4%	1.00	3.00	2.00	0.50	0.25	8

#	Field	Red = stop do not continue		Amber = more work needed or a concern, but go		Green = good to go		Total
1	The EMPRO attribute of Conceptual and measurement model: The rationale for and description of the concept and the populations that a measure is intended to assess and the relationship between these concepts: score of 85.7%	0.00%	0	0.00%	0	100.00%	8	8
2	The EMPRO attribute of Validity: The degree to which the instrument measures what it purports to measure: score of 44.4%	12.50%	1	75.00%	6	12.50%	1	8

Showing rows 1 - 2 of 2

Q21 - OES meets the discrimination OMERACT filter requirements for the outcome measure representing the Disability Domain in the core outcome set for lateral elbow tendinopathy.

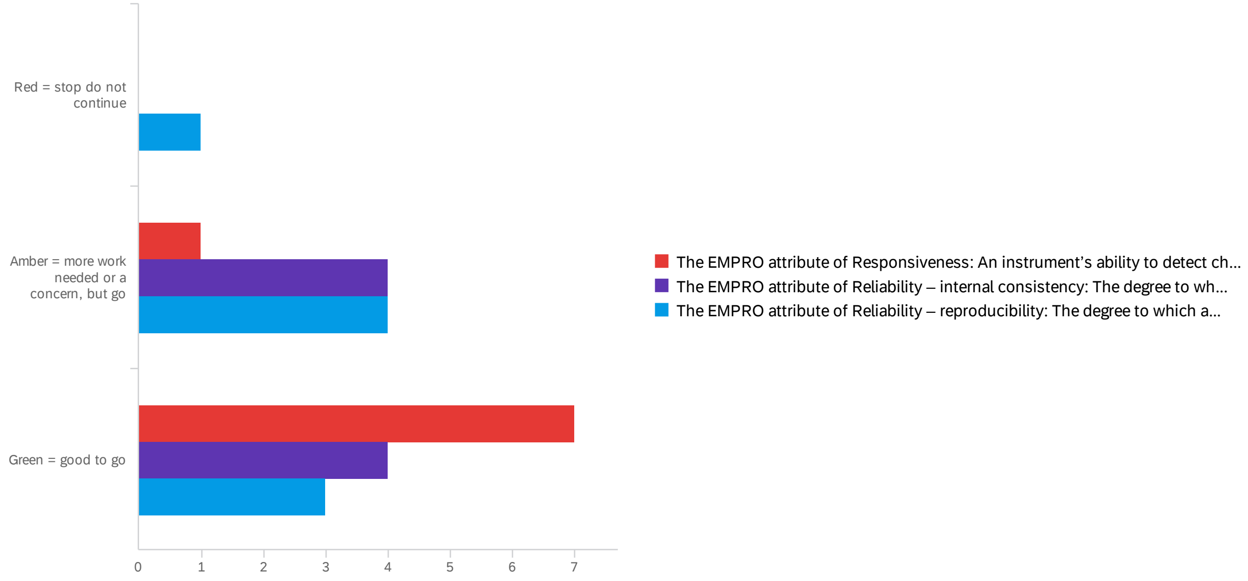

#	Field	Minimum	Maximum	Mean	Std Deviation	Variance	Count
1	The EMPRO attribute of Responsiveness: An instrument's ability to detect change over time: score of 77.8%	2.00	3.00	2.88	0.33	0.11	8
2	The EMPRO attribute of Reliability – internal consistency: The degree to which an instrument is free from random error: score of 58.3%	2.00	3.00	2.50	0.50	0.25	8
3	The EMPRO attribute of Reliability – reproducibility: The degree to which an instrument is free from random error: score of 50%	1.00	3.00	2.25	0.66	0.44	8

#	Field	Red = stop do not continue	Amber = more work needed or a concern, but go	Green = good to go	Total
1	The EMPRO attribute of Responsiveness: An instrument's ability to detect change over time: score of 77.8%	0.00% 0	12.50% 1	87.50% 7	8
2	The EMPRO attribute of Reliability – internal consistency: The degree to which an instrument is free from random error: score of 58.3%	0.00% 0	50.00% 4	50.00% 4	8

#	Field	Red = stop do not continue		Amber = more work needed or a concern, but go		Green = good to go		Total
3	The EMPRO attribute of Reliability – reproducibility: The degree to which an instrument is free from random error: score of 50%	12.50%	1	50.00%	4	37.50%	3	8

Showing rows 1 - 3 of 3

Q22 - Considering your responses to the truth and discrimination OMERACT filter above, does OES meets the requirements for the outcome measure representing the Disability Domain in the core outcome set for lateral elbow tendinopathy.

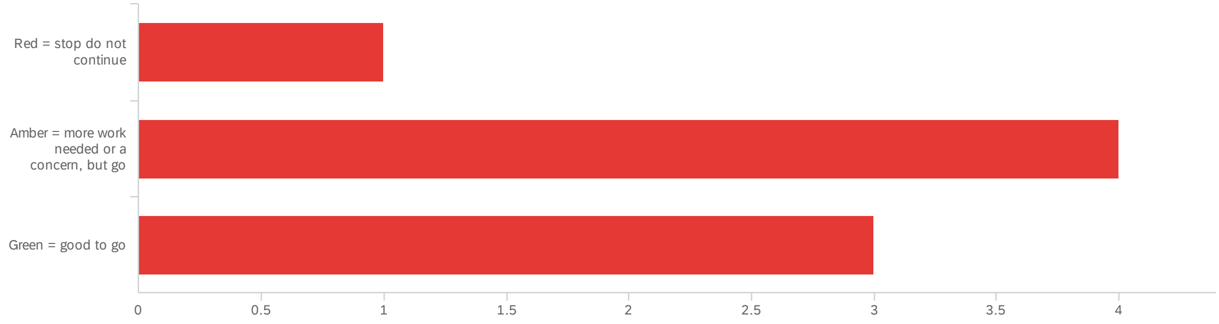

#	Field	Minimum	Maximum	Mean	Std Deviation	Variance	Count
1	The overal EMPRO Score (a composite of the above attribute scores) is: 66.59%	1.00	3.00	2.25	0.66	0.44	8

#	Field	Choice Count	
1	Red = stop do not continue	12.50%	1
2	Amber = more work needed or a concern, but go	50.00%	4
3	Green = good to go	37.50%	3
			8

Showing rows 1 - 4 of 4

Q24 - The PRTEE meets the truth OMERACT filter requirements for the outcome measure representing the Pain on Activity/Loading Domain in the core outcome set for lateral elbow tendinopathy.

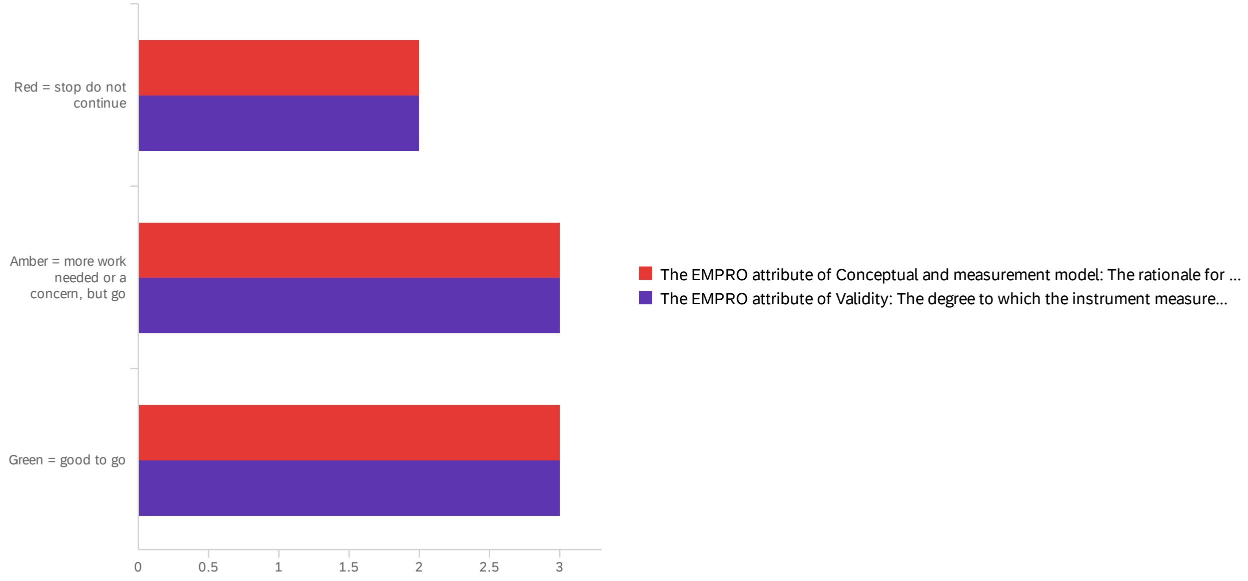

#	Field	Minimum	Maximum	Mean	Std Deviation	Variance	Count
1	The EMPRO attribute of Conceptual and measurement model: The rationale for and description of the concept and the populations that a measure is intended to assess and the relationship between these concepts: score of 42.86%	1.00	3.00	2.13	0.78	0.61	8
2	The EMPRO attribute of Validity: The degree to which the instrument measures what it purports to measure: score of 53.33%*	1.00	3.00	2.13	0.78	0.61	8

#	Field	Red = stop do not continue		Amber = more work needed or a concern, but go		Green = good to go		Total
1	The EMPRO attribute of Conceptual and measurement model: The rationale for and description of the concept and the populations that a measure is intended to assess and the relationship between these concepts: score of 42.86%	25.00%	2	37.50%	3	37.50%	3	8
2	The EMPRO attribute of Validity: The degree to which the instrument measures what it purports to measure: score of 53.33%*	25.00%	2	37.50%	3	37.50%	3	8

Showing rows 1 - 2 of 2

Q25 - PRTEE meets the discrimination OMERACT filter requirements for the outcome measure representing the Pain on Activity/Loading Domain in the core outcome set for lateral elbow tendinopathy.

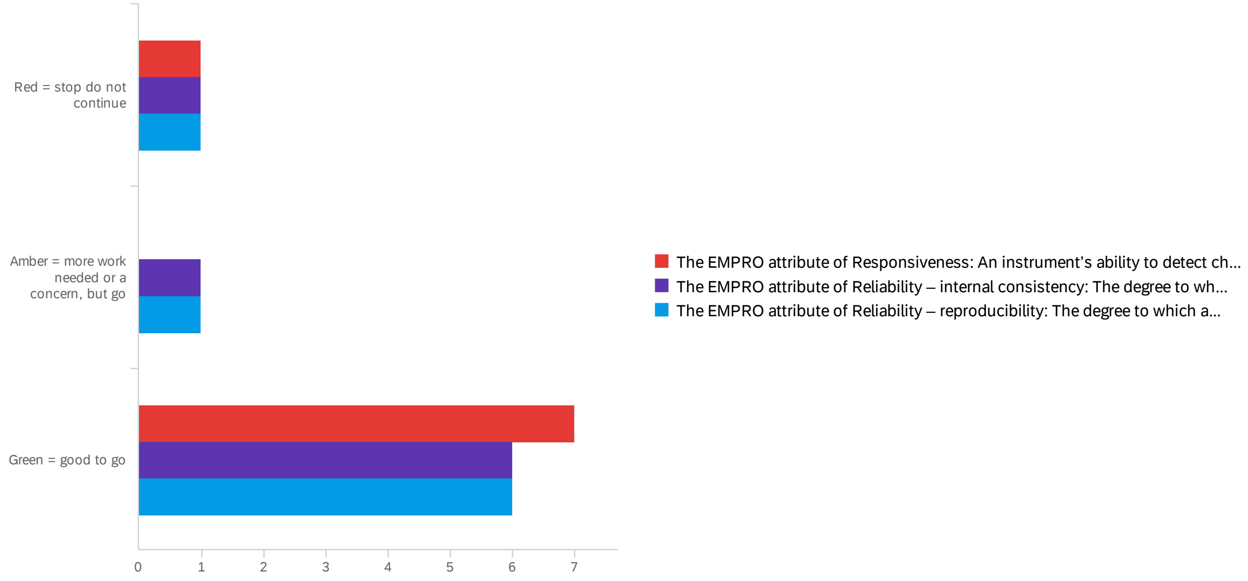

#	Field	Minimum	Maximum	Mean	Std Deviation	Variance	Count
1	The EMPRO attribute of Responsiveness: An instrument's ability to detect change over time: score of 77.78%	1.00	3.00	2.75	0.66	0.44	8
2	The EMPRO attribute of Reliability – internal consistency: The degree to which an instrument is free from random error: score of 66.67%	1.00	3.00	2.63	0.70	0.48	8
3	The EMPRO attribute of Reliability – reproducibility: The degree to which an instrument is free from random error: score of 66.67%	1.00	3.00	2.63	0.70	0.48	8

#	Field	Red = stop do not continue	Amber = more work needed or a concern, but go	Green = good to go	Total
1	The EMPRO attribute of Responsiveness: An instrument's ability to detect change over time: score of 77.78%	12.50% 1	0.00% 0	87.50% 7	8
2	The EMPRO attribute of Reliability – internal consistency: The degree to which an instrument is free from random error: score of 66.67%	12.50% 1	12.50% 1	75.00% 6	8

#	Field	Red = stop do not continue		Amber = more work needed or a concern, but go		Green = good to go		Total
3	The EMPRO attribute of Reliability – reproducibility: The degree to which an instrument is free from random error: score of 66.67%	12.50%	1	12.50%	1	75.00%	6	8

Showing rows 1 - 3 of 3

Q26 - Considering your responses to the truth and discrimination OMERACT filter above and the fact that the EMPRO scores will overinflate the validity and overall score due to the lack of validity data for the pain subscale, does PRTEE meets the requirements for the outcome measure representing the Pain on Activity/Loading Domain in the core outcome set for lateral elbow tendinopathy.

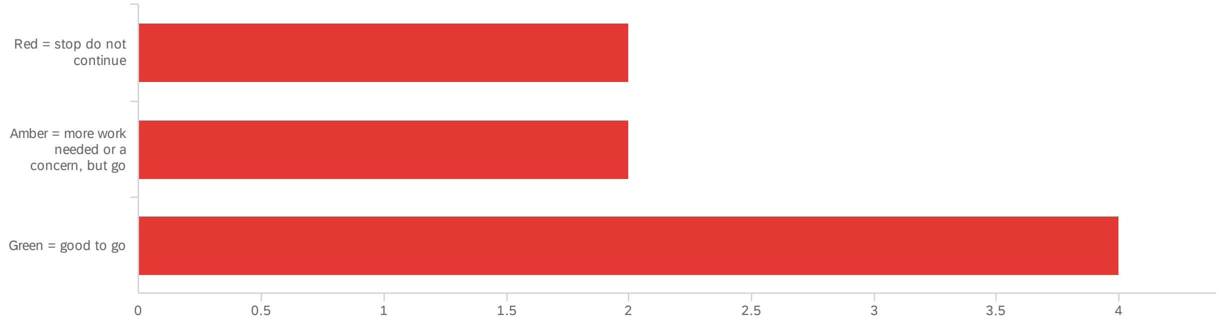

#	Field	Minimum	Maximum	Mean	Std Deviation	Variance	Count
1	The overal EMPRO Score (a composite of the above attribute scores) is: 57.02%*	1.00	3.00	2.25	0.83	0.69	8

#	Field	Choice Count	
1	Red = stop do not continue	25.00%	2
2	Amber = more work needed or a concern, but go	25.00%	2
3	Green = good to go	50.00%	4
			8

Showing rows 1 - 4 of 4

Q28 - The TEFS meets the truth OMERACT filter requirements for the outcome measure representing the Pain on Activity/Loading Domain in the core outcome set for lateral elbow tendinopathy.

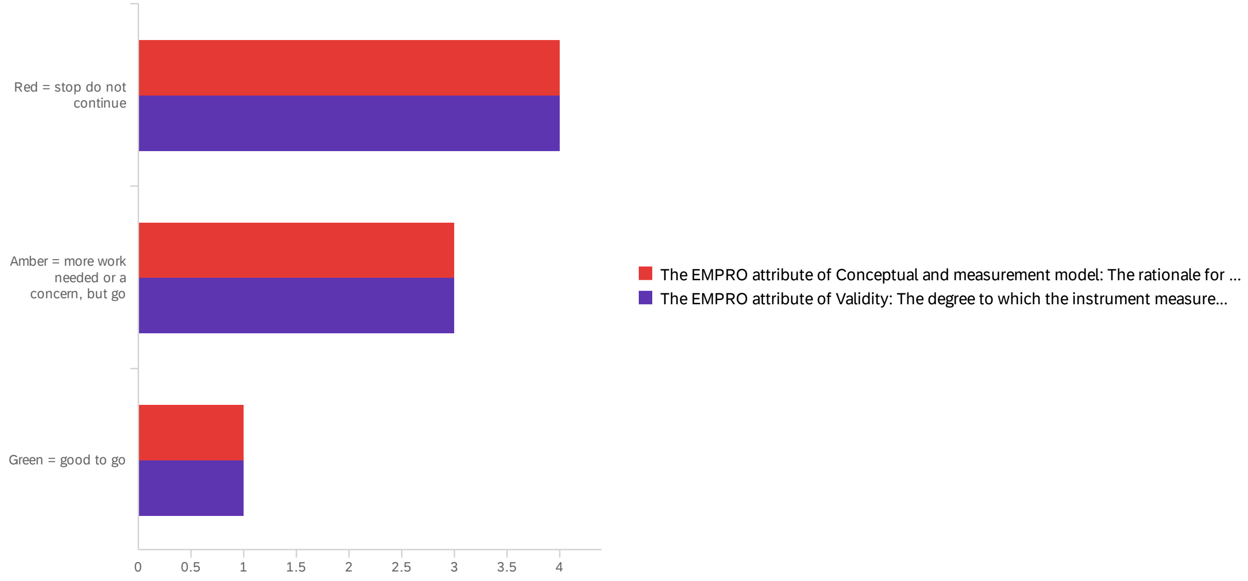

#	Field	Minimum	Maximum	Mean	Std Deviation	Variance	Count
1	The EMPRO attribute of Conceptual and measurement model: The rationale for and description of the concept and the populations that a measure is intended to assess and the relationship between these concepts: score of 33.3%	1.00	3.00	1.63	0.70	0.48	8
2	The EMPRO attribute of Validity: The degree to which the instrument measures what it purports to measure: score of 33.3%	1.00	3.00	1.63	0.70	0.48	8

#	Field	Red = stop do not continue		Amber = more work needed or a concern, but go		Green = good to go		Total
1	The EMPRO attribute of Conceptual and measurement model: The rationale for and description of the concept and the populations that a measure is intended to assess and the relationship between these concepts: score of 33.3%	50.00%	4	37.50%	3	12.50%	1	8
2	The EMPRO attribute of Validity: The degree to which the instrument measures what it purports to measure: score of 33.3%	50.00%	4	37.50%	3	12.50%	1	8

Showing rows 1 - 2 of 2

Q29 - TEFS meets the discrimination OMERACT filter requirements for the outcome measure representing the Pain on Activity/Loading Domain in the core outcome set for lateral elbow tendinopathy.

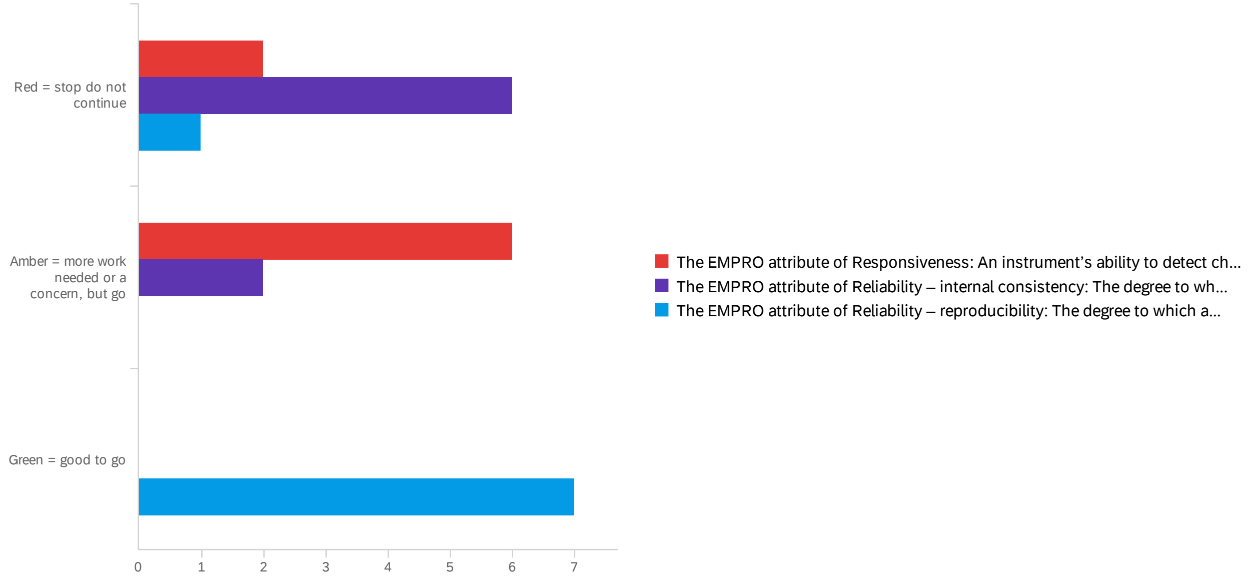

#	Field	Minimum	Maximum	Mean	Std Deviation	Variance	Count
1	The EMPRO attribute of Responsiveness: An instrument's ability to detect change over time: score of 44.4%	1.00	2.00	1.75	0.43	0.19	8
2	The EMPRO attribute of Reliability – internal consistency: The degree to which an instrument is free from random error: score of 8.3%	1.00	2.00	1.25	0.43	0.19	8
3	The EMPRO attribute of Reliability – reproducibility: The degree to which an instrument is free from random error: score of 75%	1.00	3.00	2.75	0.66	0.44	8

#	Field	Red = stop do not continue	Amber = more work needed or a concern, but go	Green = good to go	Total
1	The EMPRO attribute of Responsiveness: An instrument's ability to detect change over time: score of 44.4%	25.00% 2	75.00% 6	0.00% 0	8
2	The EMPRO attribute of Reliability – internal consistency: The degree to which an instrument is free from random error: score of 8.3%	75.00% 6	25.00% 2	0.00% 0	8

#	Field	Red = stop do not continue		Amber = more work needed or a concern, but go		Green = good to go		Total
3	The EMPRO attribute of Reliability – reproducibility: The degree to which an instrument is free from random error: score of 75%	12.50%	1	0.00%	0	87.50%	7	8

Showing rows 1 - 3 of 3

Q30 - Considering your responses to the truth and discrimination OMERACT filter above, does TEFS meet the requirements for the outcome measure representing the Pain on Activity/Loading Domain in the core outcome set for lateral elbow tendinopathy.

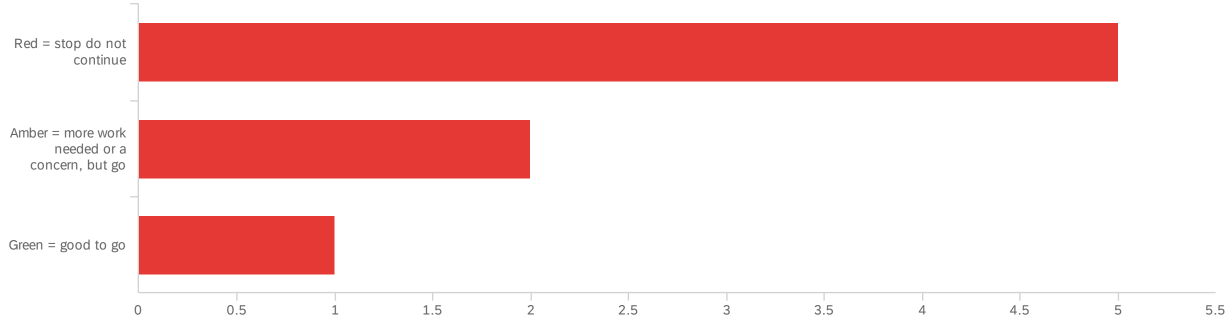

#	Field	Minimum	Maximum	Mean	Std Deviation	Variance	Count
1	The overal EMPRO Score (a composite of the above attribute scores) is: 41.67%	1.00	3.00	1.50	0.71	0.50	8

#	Field	Choice Count	
1	Red = stop do not continue	62.50%	5
2	Amber = more work needed or a concern, but go	25.00%	2
3	Green = good to go	12.50%	1
			8

Showing rows 1 - 4 of 4

Q36 - The TEFS meets the truth OMERACT filter requirements for the outcome measure representing the Pain over a Specified Time Domain in the core outcome set for lateral elbow tendinopathy.

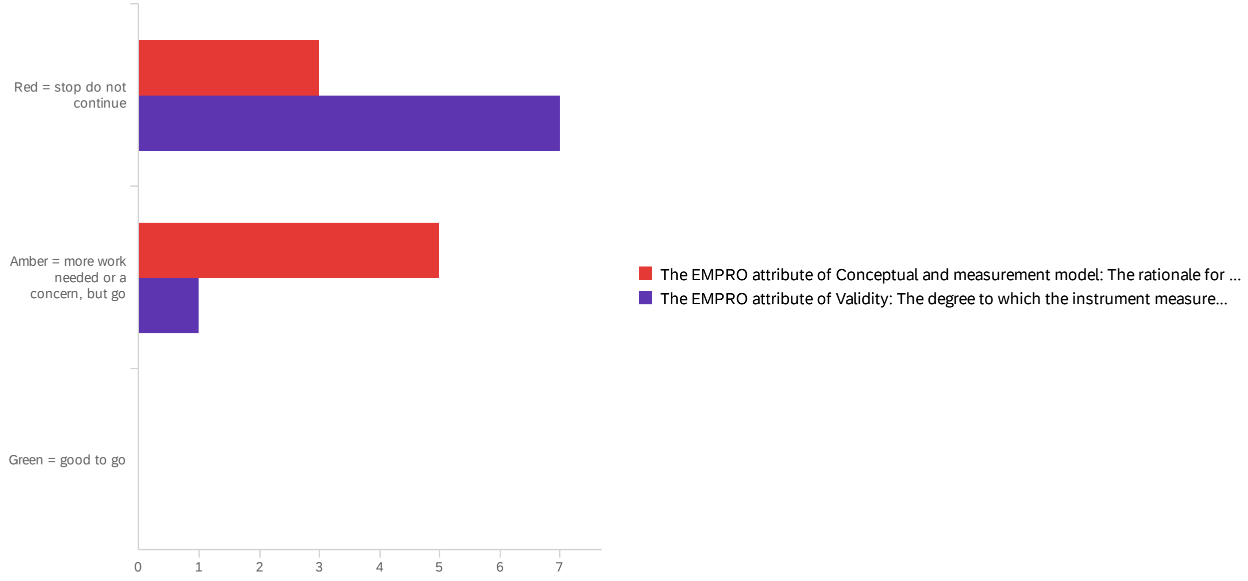

#	Field	Minimum	Maximum	Mean	Std Deviation	Variance	Count
1	The EMPRO attribute of Conceptual and measurement model: The rationale for and description of the concept and the populations that a measure is intended to assess and the relationship between these concepts: score of 33.3%	1.00	2.00	1.63	0.48	0.23	8
2	The EMPRO attribute of Validity: The degree to which the instrument measures what it purports to measure: score of 33.3%	1.00	2.00	1.13	0.33	0.11	8

#	Field	Red = stop do not continue		Amber = more work needed or a concern, but go		Green = good to go		Total
1	The EMPRO attribute of Conceptual and measurement model: The rationale for and description of the concept and the populations that a measure is intended to assess and the relationship between these concepts: score of 33.3%	37.50%	3	62.50%	5	0.00%	0	8
2	The EMPRO attribute of Validity: The degree to which the instrument measures what it purports to measure: score of 33.3%	87.50%	7	12.50%	1	0.00%	0	8

Showing rows 1 - 2 of 2

Q37 - TEFS meets the discrimination OMERACT filter requirements for the outcome measure representing the Pain over a Specified Time Domain in the core outcome set for lateral elbow tendinopathy.

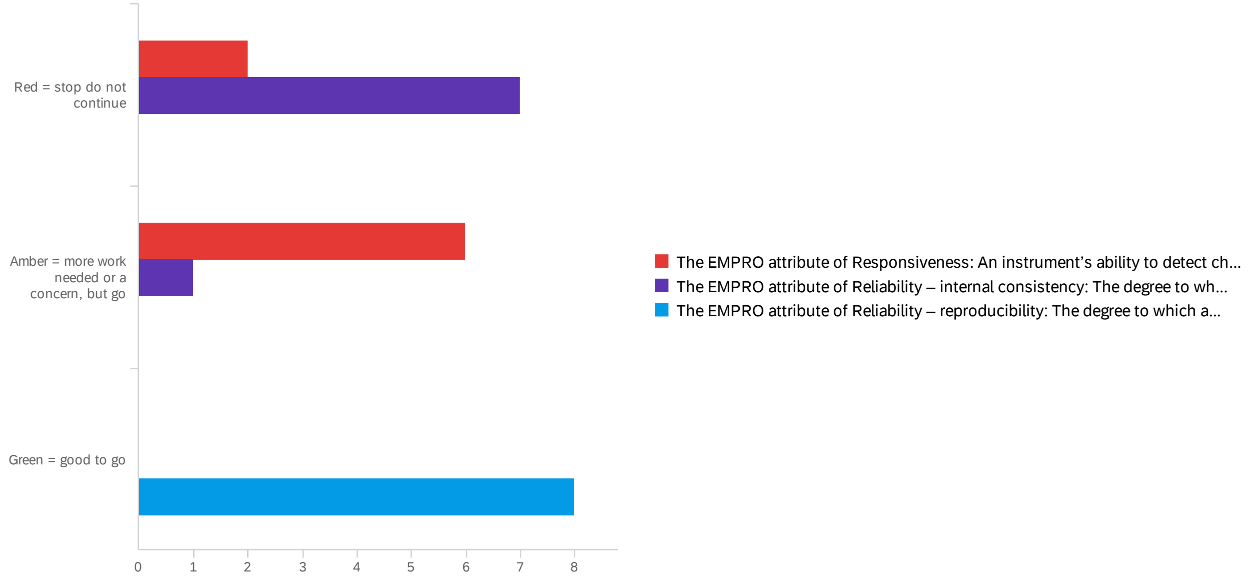

#	Field	Minimum	Maximum	Mean	Std Deviation	Variance	Count
1	The EMPRO attribute of Responsiveness: An instrument's ability to detect change over time: score of 44.4%	1.00	2.00	1.75	0.43	0.19	8
2	The EMPRO attribute of Reliability – internal consistency: The degree to which an instrument is free from random error: score of 8.3%	1.00	2.00	1.13	0.33	0.11	8
3	The EMPRO attribute of Reliability – reproducibility: The degree to which an instrument is free from random error: score of 75%	3.00	3.00	3.00	0.00	0.00	8

#	Field	Red = stop do not continue	Amber = more work needed or a concern, but go	Green = good to go	Total
1	The EMPRO attribute of Responsiveness: An instrument's ability to detect change over time: score of 44.4%	25.00% 2	75.00% 6	0.00% 0	8
2	The EMPRO attribute of Reliability – internal consistency: The degree to which an instrument is free from random error: score of 8.3%	87.50% 7	12.50% 1	0.00% 0	8

#	Field	Red = stop do not continue		Amber = more work needed or a concern, but go		Green = good to go		Total
3	The EMPRO attribute of Reliability – reproducibility: The degree to which an instrument is free from random error: score of 75%	0.00%	0	0.00%	0	100.00%	8	8

Showing rows 1 - 3 of 3

Q38 - Considering your responses to the truth and discrimination OMERACT filter above, does TEFS meet the requirements for the outcome measure representing the Pain over a Specified Time Domain in the core outcome set for lateral elbow tendinopathy.

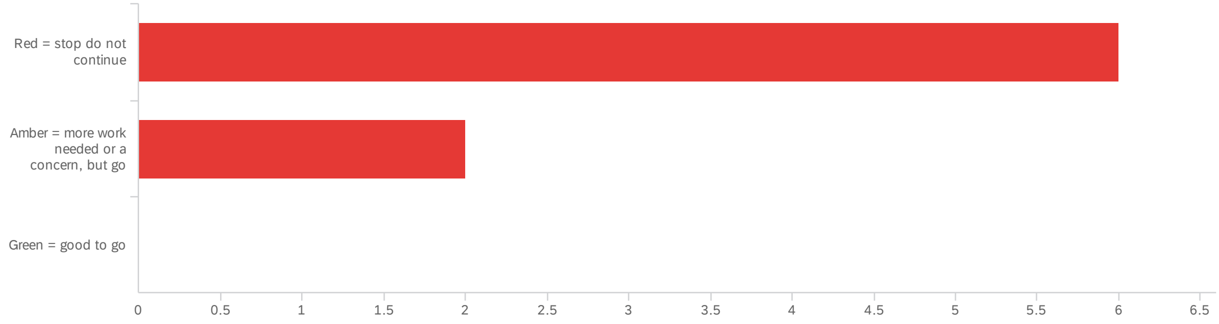

#	Field	Minimum	Maximum	Mean	Std Deviation	Variance	Count
1	The overal EMPRO Score (a composite of the above attribute scores) is: 41.67%	1.00	2.00	1.25	0.43	0.19	8

#	Field	Choice Count	
1	Red = stop do not continue	75.00%	6
2	Amber = more work needed or a concern, but go	25.00%	2
3	Green = good to go	0.00%	0
			8

Showing rows 1 - 4 of 4

Q32 - The PRTEE meets the truth OMERACT filter requirements for the outcome measure representing the Function Domain in the core outcome set for lateral elbow tendinopathy.

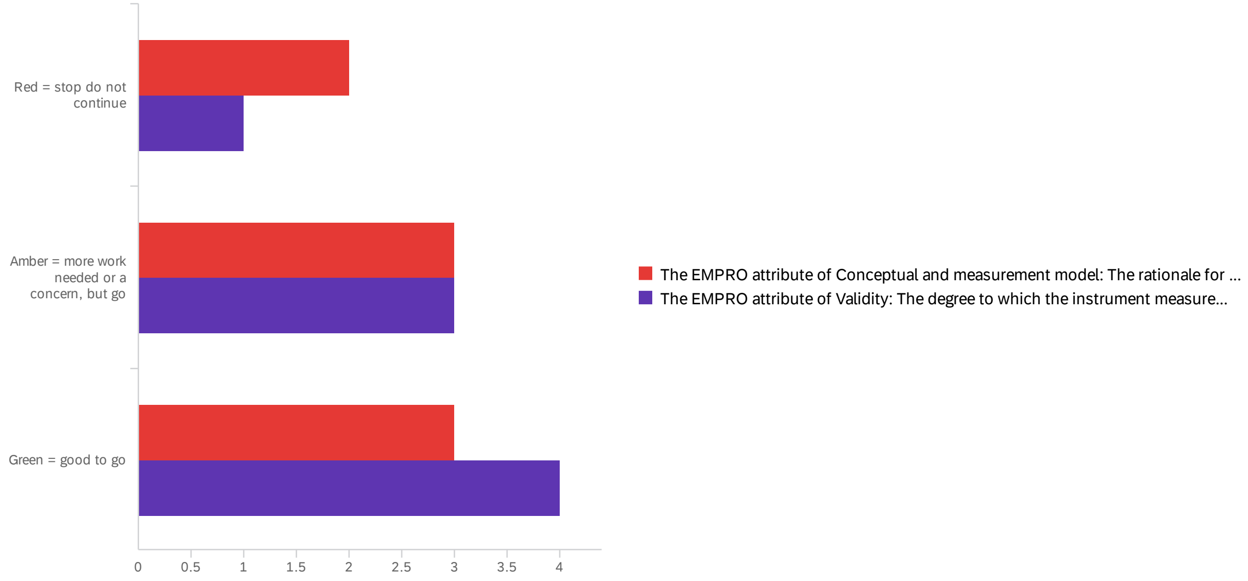

#	Field	Minimum	Maximum	Mean	Std Deviation	Variance	Count
1	The EMPRO attribute of Conceptual and measurement model: The rationale for and description of the concept and the populations that a measure is intended to assess and the relationship between these concepts: score of 42.86%	1.00	3.00	2.13	0.78	0.61	8
2	The EMPRO attribute of Validity: The degree to which the instrument measures what it purports to measure: score of 53.33%*	1.00	3.00	2.38	0.70	0.48	8

#	Field	Red = stop do not continue		Amber = more work needed or a concern, but go		Green = good to go		Total
1	The EMPRO attribute of Conceptual and measurement model: The rationale for and description of the concept and the populations that a measure is intended to assess and the relationship between these concepts: score of 42.86%	25.00%	2	37.50%	3	37.50%	3	8
2	The EMPRO attribute of Validity: The degree to which the instrument measures what it purports to measure: score of 53.33%*	12.50%	1	37.50%	3	50.00%	4	8

Showing rows 1 - 2 of 2

Q33 - PRTEE meets the discrimination OMERACT filter requirements for the outcome measure representing the Function Domain in the core outcome set for lateral elbow tendinopathy.

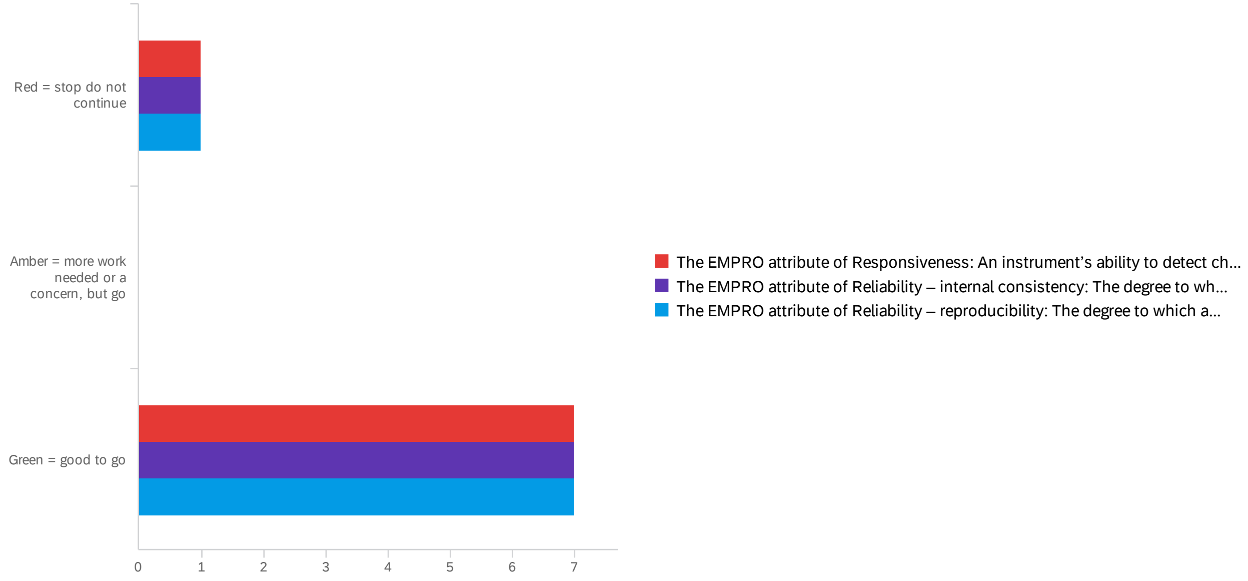

#	Field	Minimum	Maximum	Mean	Std Deviation	Variance	Count
1	The EMPRO attribute of Responsiveness: An instrument's ability to detect change over time: score of 77.78%	1.00	3.00	2.75	0.66	0.44	8
2	The EMPRO attribute of Reliability – internal consistency: The degree to which an instrument is free from random error: score of 66.67%	1.00	3.00	2.75	0.66	0.44	8
3	The EMPRO attribute of Reliability – reproducibility: The degree to which an instrument is free from random error: score of 66.67%	1.00	3.00	2.75	0.66	0.44	8

#	Field	Red = stop do not continue	Amber = more work needed or a concern, but go	Green = good to go	Total
1	The EMPRO attribute of Responsiveness: An instrument's ability to detect change over time: score of 77.78%	12.50% 1	0.00% 0	87.50% 7	8
2	The EMPRO attribute of Reliability – internal consistency: The degree to which an instrument is free from random error: score of 66.67%	12.50% 1	0.00% 0	87.50% 7	8

#	Field	Red = stop do not continue		Amber = more work needed or a concern, but go		Green = good to go		Total
3	The EMPRO attribute of Reliability – reproducibility: The degree to which an instrument is free from random error: score of 66.67%	12.50%	1	0.00%	0	87.50%	7	8

Showing rows 1 - 3 of 3

Q34 - Considering your responses to the truth and discrimination OMERACT filter above and the fact that the EMPRO scores will overinflate the validity and overall score due to the lack of validity data for the function subscale, does PRTEE meets the requirements for the outcome measure representing the Function Domain in the core outcome set for lateral elbow tendinopathy.

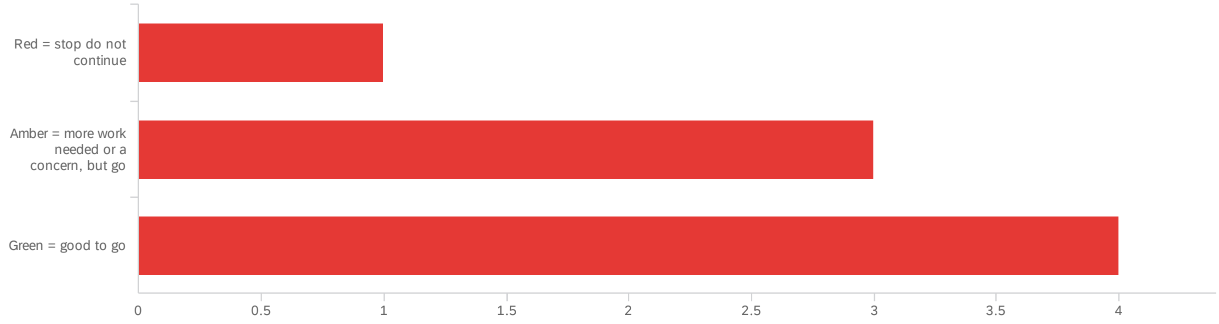

#	Field	Minimum	Maximum	Mean	Std Deviation	Variance	Count
1	The overal EMPRO Score (a composite of the above attribute scores) is: 57.02%*	1.00	3.00	2.38	0.70	0.48	8

#	Field	Choice Count	
1	Red = stop do not continue	12.50%	1
2	Amber = more work needed or a concern, but go	37.50%	3
3	Green = good to go	50.00%	4
			8

Showing rows 1 - 4 of 4

Q40 - The MGS meets the truth OMERACT filter requirements for the outcome measure representing the Physical Function Capacity Domain in the core outcome set for lateral elbow tendinopathy.

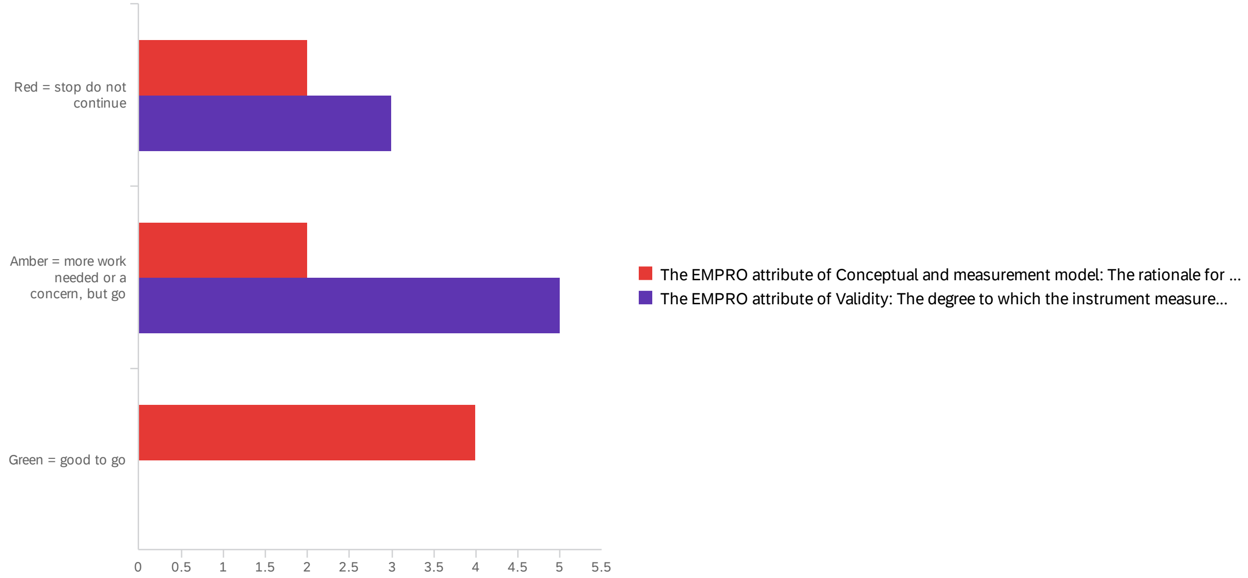

#	Field	Minimum	Maximum	Mean	Std Deviation	Variance	Count
1	The EMPRO attribute of Conceptual and measurement model: The rationale for and description of the concept and the populations that a measure is intended to assess and the relationship between these concepts: score of 47.62%	1.00	3.00	2.25	0.83	0.69	8
2	The EMPRO attribute of Validity: The degree to which the instrument measures what it purports to measure: score of 0%	1.00	2.00	1.63	0.48	0.23	8

#	Field	Red = stop do not continue		Amber = more work needed or a concern, but go		Green = good to go		Total
1	The EMPRO attribute of Conceptual and measurement model: The rationale for and description of the concept and the populations that a measure is intended to assess and the relationship between these concepts: score of 47.62%	25.00%	2	25.00%	2	50.00%	4	8
2	The EMPRO attribute of Validity: The degree to which the instrument measures what it purports to measure: score of 0%	37.50%	3	62.50%	5	0.00%	0	8

Showing rows 1 - 2 of 2

Q41 - MGS meets the discrimination OMERACT filter requirements for the outcome measure representing the Physical Function Capacity Domain in the core outcome set for lateral elbow tendinopathy.

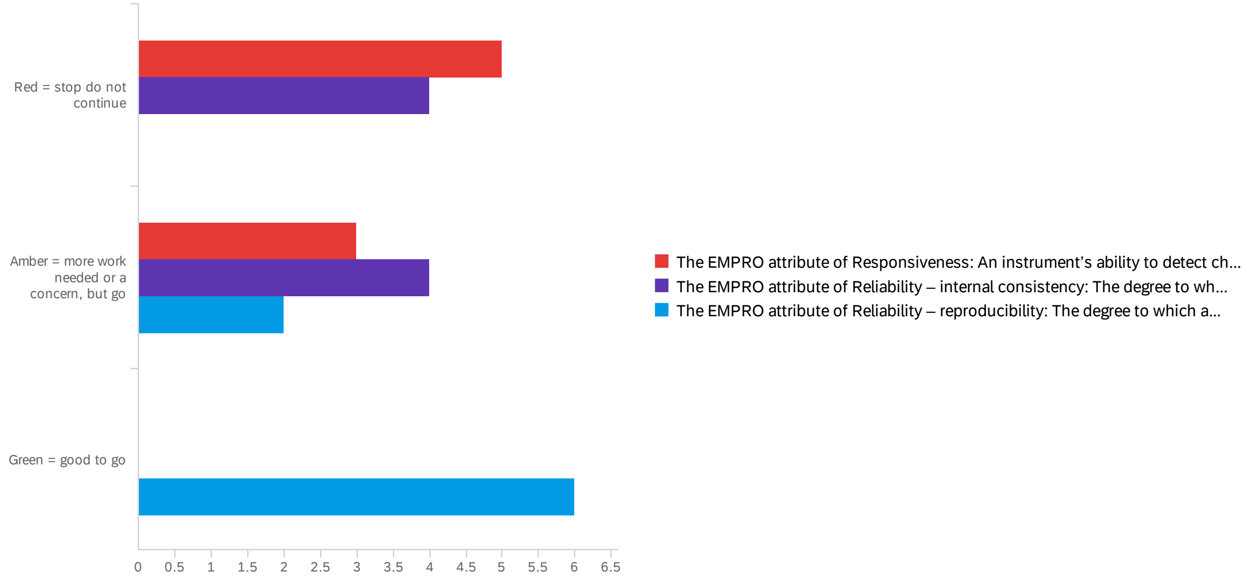

#	Field	Minimum	Maximum	Mean	Std Deviation	Variance	Count
1	The EMPRO attribute of Responsiveness: An instrument's ability to detect change over time: score of 22.22%	1.00	2.00	1.38	0.48	0.23	8
2	The EMPRO attribute of Reliability – internal consistency: The degree to which an instrument is free from random error: score of 0%	1.00	2.00	1.50	0.50	0.25	8
3	The EMPRO attribute of Reliability – reproducibility: The degree to which an instrument is free from random error: score of 75%	2.00	3.00	2.75	0.43	0.19	8

#	Field	Red = stop do not continue	Amber = more work needed or a concern, but go	Green = good to go	Total
1	The EMPRO attribute of Responsiveness: An instrument's ability to detect change over time: score of 22.22%	62.50% 5	37.50% 3	0.00% 0	8
2	The EMPRO attribute of Reliability – internal consistency: The degree to which an instrument is free from random error: score of 0%	50.00% 4	50.00% 4	0.00% 0	8

#	Field	Red = stop do not continue		Amber = more work needed or a concern, but go		Green = good to go		Total
3	The EMPRO attribute of Reliability – reproducibility: The degree to which an instrument is free from random error: score of 75%	0.00%	0	25.00%	2	75.00%	6	8

Showing rows 1 - 3 of 3

Q42 - Considering your responses to the truth and discrimination OMERACT filter above and the fact that the EMPRO scores will overinflate the validity and overall score due to the lack of validity data for the function subscale, does MGS meets the requirements for the outcome measure representing the Physical Function Capacity Domain in the core outcome set for lateral elbow tendinopathy.

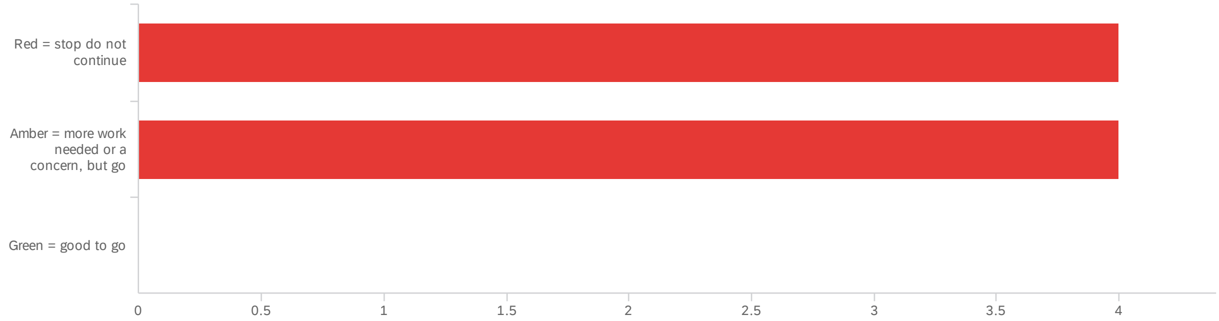

#	Field	Minimum	Maximum	Mean	Std Deviation	Variance	Count
1	The overal EMPRO Score (a composite of the above attribute scores) is: 25.08%	1.00	2.00	1.50	0.50	0.25	8

#	Field	Choice Count	
1	Red = stop do not continue	50.00%	4
2	Amber = more work needed or a concern, but go	50.00%	4
3	Green = good to go	0.00%	0
			8

Showing rows 1 - 4 of 4

Q44 - The PFG meets the truth OMERACT filter requirements for the outcome measure representing the Physical Function Capacity Domain in the core outcome set for lateral elbow tendinopathy.

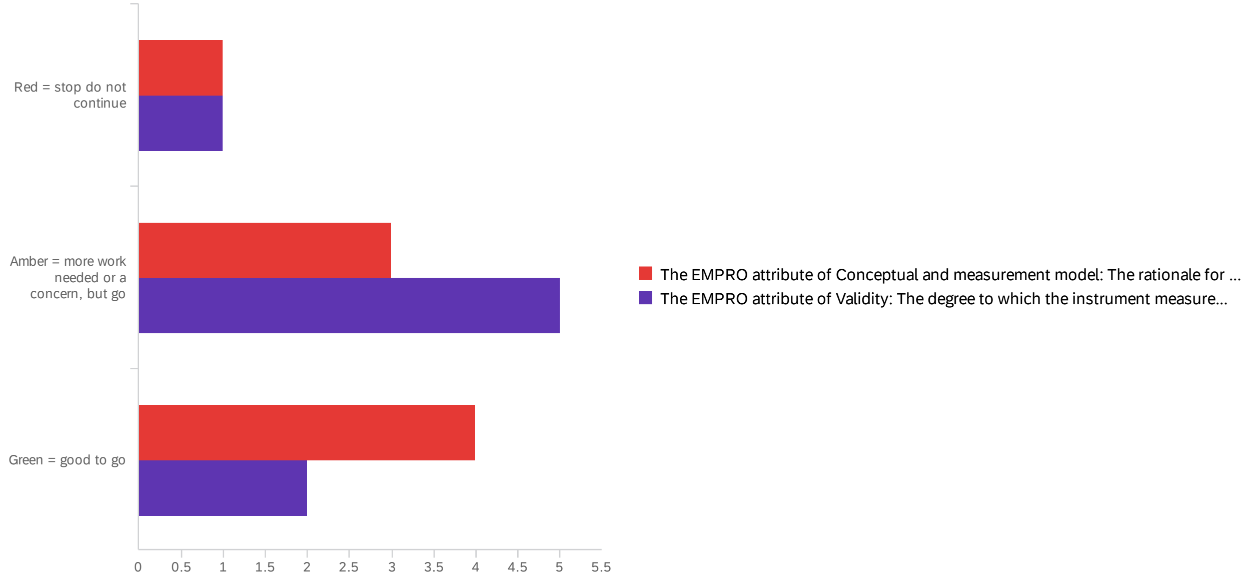

#	Field	Minimum	Maximum	Mean	Std Deviation	Variance	Count
1	The EMPRO attribute of Conceptual and measurement model: The rationale for and description of the concept and the populations that a measure is intended to assess and the relationship between these concepts: score of 47.62%	1.00	3.00	2.38	0.70	0.48	8
2	The EMPRO attribute of Validity: The degree to which the instrument measures what it purports to measure: score of 38.89%	1.00	3.00	2.13	0.60	0.36	8

#	Field	Red = stop do not continue		Amber = more work needed or a concern, but go		Green = good to go		Total
1	The EMPRO attribute of Conceptual and measurement model: The rationale for and description of the concept and the populations that a measure is intended to assess and the relationship between these concepts: score of 47.62%	12.50%	1	37.50%	3	50.00%	4	8
2	The EMPRO attribute of Validity: The degree to which the instrument measures what it purports to measure: score of 38.89%	12.50%	1	62.50%	5	25.00%	2	8

Showing rows 1 - 2 of 2

Q45 - PFG meets the discrimination OMERACT filter requirements for the outcome measure representing the Physical Function Capacity Domain in the core outcome set for lateral elbow tendinopathy.

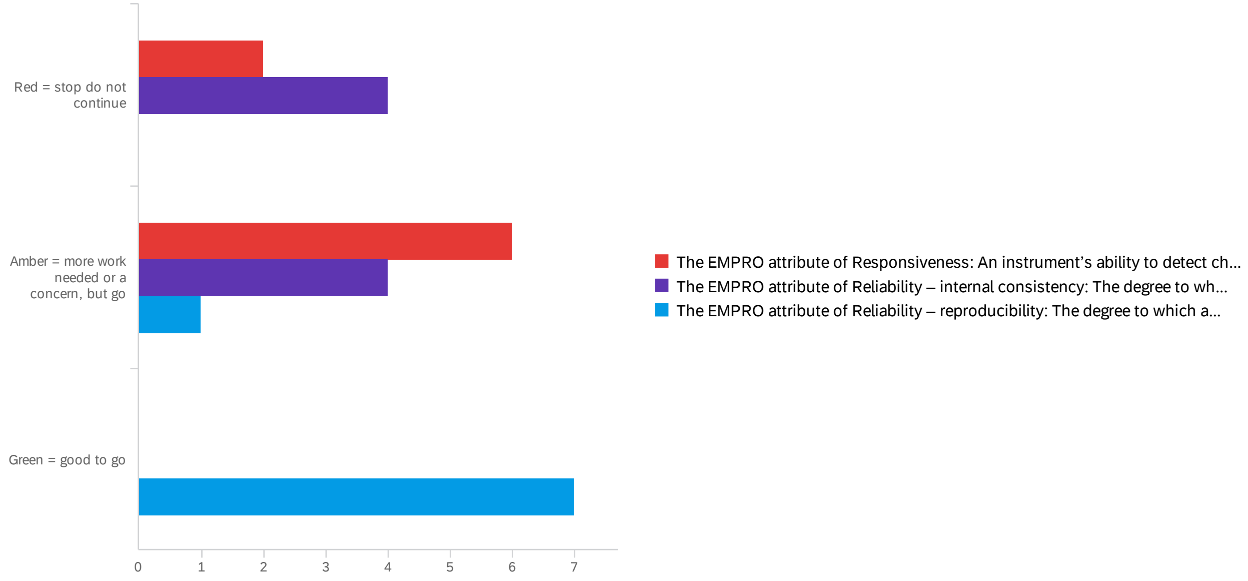

#	Field	Minimum	Maximum	Mean	Std Deviation	Variance	Count
1	The EMPRO attribute of Responsiveness: An instrument's ability to detect change over time: score of 22.22%	1.00	2.00	1.75	0.43	0.19	8
2	The EMPRO attribute of Reliability – internal consistency: The degree to which an instrument is free from random error: score of 0%	1.00	2.00	1.50	0.50	0.25	8
3	The EMPRO attribute of Reliability – reproducibility: The degree to which an instrument is free from random error: score of 75%	2.00	3.00	2.88	0.33	0.11	8

#	Field	Red = stop do not continue	Amber = more work needed or a concern, but go	Green = good to go	Total
1	The EMPRO attribute of Responsiveness: An instrument's ability to detect change over time: score of 22.22%	25.00% 2	75.00% 6	0.00% 0	8
2	The EMPRO attribute of Reliability – internal consistency: The degree to which an instrument is free from random error: score of 0%	50.00% 4	50.00% 4	0.00% 0	8

#	Field	Red = stop do not continue		Amber = more work needed or a concern, but go		Green = good to go		Total
3	The EMPRO attribute of Reliability – reproducibility: The degree to which an instrument is free from random error: score of 75%	0.00%	0	12.50%	1	87.50%	7	8

Showing rows 1 - 3 of 3

Q46 - Considering your responses to the truth and discrimination OMERACT filter above and the fact that the EMPRO scores will overinflate the validity and overall score due to the lack of validity data for the function subscale, does PFG meets the requirements for the outcome measure representing the Physical Function Capacity Domain in the core outcome set for lateral elbow tendinopathy.

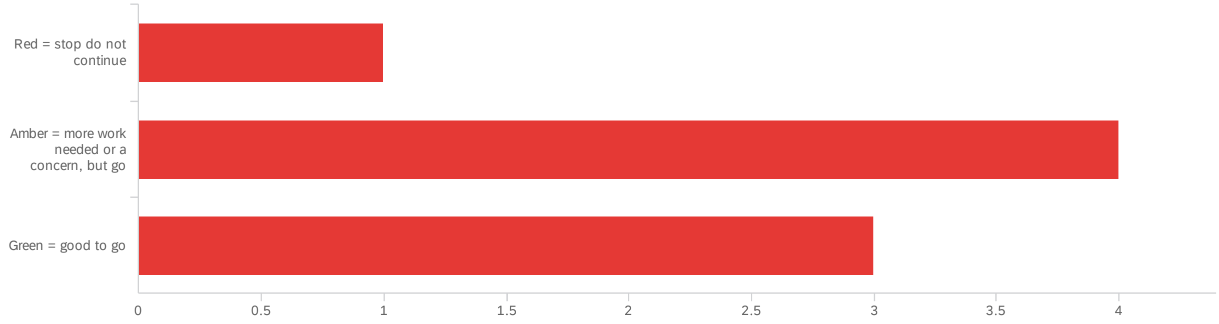

#	Field	Minimum	Maximum	Mean	Std Deviation	Variance	Count
1	The overal EMPRO Score (a composite of the above attribute scores) is: 32.86%	1.00	3.00	2.25	0.66	0.44	8

#	Field	Choice Count	
1	Red = stop do not continue	12.50%	1
2	Amber = more work needed or a concern, but go	50.00%	4
3	Green = good to go	37.50%	3
			8

Showing rows 1 - 4 of 4

End of Report
