# Supplementary material for: Development of a core outcome set for lateral elbow tendinopathy (COS-LET) using best available evidence and an international consensus process
Source: Br J Sports Med. 2022 Feb 8;56(12):657–66. doi: 10.1136/bjsports-2021-105044 (PMC9163713; doi:10.1136/bjsports-2021-105044)
Supplement: Supplementary data [file bjsports-2021-105044supp003.pdf]

Delphi Process for Core Outcome Set for Lateral Elbow Tendinopathy (COS-LET): Report of findings from round 1 and 2 Surveys plus input from patient focus groups

In brief, we have now undertaken two surveys and patient focus groups on the matter of what outcome measures will be in the COS-LET. This report contains the results of this process to date – which will be the focus of our forthcoming consensus meeting. As well as the report below, we have attached an agenda, the domains paper, the first survey report (which you received with the second survey) for your reference, and a folder containing the outcome measures that we will be discussing at our consensus meeting.

Context:

1. There is a high level of heterogeneity in outcome measures used in trials of lateral elbow tendinopathy (LET), which makes evidence synthesis across studies difficult.
2. Previous work in the field of tendinopathy has established through a consensus exercise nine core health-related domains that should be measured in tendinopathy research.
3. The aim of this study is to develop a Core Outcome Set for Lateral Elbow Tendinopathy (COS-LET) mapping to these core domains.

Methods:

The development of the COS-LET is being developed as per the following process:

1. Systematic review of studies investigating LET has revealed a comprehensive list of all instruments that have previously been used to quantify treatment effect or outcome.
2. These instruments were matched to the list of nine core tendinopathy outcome domains by a Steering Committee of clinicians and researchers with a specialist interest in LET resulting in a set of candidate instruments.
3. You then responded to the first survey that asked you about the outcome measures for each domain. Seven patients also completed the first survey.
4. The committee then collated your responses, systematically reviewed the clinimetric/psychometric literature and rated each instrument using the EMPRO score – this information was then included in the second survey you completed.
5. You then responded to the second survey to determine what measures will be in the COS-LET, and also what may we consider in the interim (for those measures that did not make it into the COS-LET).
6. The committee then collated your responses and presented these results to two focus group meetings with some patients in the UK and Australia.
7. The results of your survey responses and the patient focus groups have now been collated and are now presented to you herein in the lead up to our consensus meeting. The report from the first survey is also appended for your reference.

Results (of second survey and patient focus group):

The results of the second survey of healthcare professionals and that of the patient focus groups are shown herein in Table 1 and 2. **Table 1** shows the characteristics of the healthcare professionals and patients participating in this consensus process. **Table 2** is a snapshot of the results of the second survey and the patient focus group meetings.

In summary, there was only one outcome that was considered (voted) to be in the core outcome set for lateral elbow tendinopathy (COS-LET) – the Patient Rated Tennis Elbow

Evaluation (PRTEE) for the Disability domain – and patients agreed. This will be ratified at our consensus meeting.

This then leaves us to make some decisions about which, if any, measures we will recommend as interim measures for the remaining domains – and importantly to plan for ongoing work in developing the COS-LET. To this end, there was agreement for PRTEE (some items on the pain subscale) to be used in the interim as a measure of pain on activity/loading domain. This was also the case for the function domain – PRTEE function subscale – though one group of patients (AUS) indicated that some of the items may not cover their specific issues and that other activities/functions may be more relevant to their specific case. We plan to commence the meeting with discussion about the PRTEE as an interim measure for the pain and function domains.

As you can see in Table 2 there are 4 domains that have no clinimetric properties and some discordance with survey results and patient views – this will be a focus of discussions at the consensus meeting. An exception to this is grip strength in that it did have some clinimetric information to consider, but discordant views between survey response and patients.

Table 1: Participant Characteristics (n (%) unless otherwise stated) of those who completed the full survey and provided these details (39 participants commenced, but 2 did not complete). UK focus group patients were a sub-group of those who completed the survey, whereas the AUS patient sub-group had not completed the survey.

Characteristics	Healthcare Professionals (N=37)	Patients (N=7)	AUS focus group patients (N=2)	UK focus group patients (N=3)
Sex: Male	25 (67.6)	2 (28.6)	2 (100)	1 (33)
Age: median (IQR; min-max) years	51 (43-57; 34-68)	48 (47.5-54.5; 26-59)	36.5 (36-37)	51.7 (48-59)
Role:				
Clinician	2 (5.4)			
Researcher	5 (13.5)			
Clinician Researcher	30 (81.1)			
Not a Clinician or Researcher		7 (100)	2 (100)	3 (100)
Highest academic qualification:				
PhD	21 (56.8)			
Master	6 (16.2)	2 (28.6)		1 (33)
Doctor of Medicine	6 (16.2)			
Postgraduate Diploma/Certificate			1 (50)	
Bachelor	3 (8.1)	3 (42.9)	1 (50)	1 (33)
Undergraduate Diploma/Certificate		1 (14.3)		1 (33)
Not specified	1 (2.7)			
No university qualification		1 (14.3)		
Profession:				
Physiotherapist	16 (43.2)			
Orthopaedic surgeon	14 (37.8)			
Sports & Exercise Medicine Physician	3 (8.1)			
Not specified	3 (8.1)			
Rheumatologist	1 (2.7)			
Patient		7 (100%)	2 (100)	3 (100)
Lateral elbow tendinopathy:				
Current history	1 (2.7)	5 (71.4)	2 (100)	1 (33)
Past history	10 (27.0)	4 (57.1)		2 (67)
Country where work:				
Australia	11 (29.7)	2 (28.6)	2 (100)	
United Kingdom	10 (27.0)	5 (71.4)		3 (100)
USA	3 (8.1)			
Canada and Norway each:	2 (5.4)			

Belgium, Finland, Greece, Israel, Italy, Netherlands, Spain, Sweden, and Turkey each:	1 (2.7)

Table 2: Summary of second survey and patient focus groups. [Note: Strong message to avoid over-burdening with too many questionnaires (UK patients)]

Insufficient psychometric evidence / In COS-LET / Interim suggestion / Interim suggestion - patients / Unsure / Not in COS-LET	**In COS-LET?** Yes	No	Unsure	**Interim Suggestion?** Yes	No	UK Focus Group (n=3/5)	UK Comments	AUS Focus Group (n=2/4)	AUS comments
**Disability**									
DASH	8.11	67.57	24.32						
Oxford Elbow Score	16.22	51.35	32.43						
PRTEE (Patient Rated Tennis Elbow Evaluation)	70.27	13.52	16.22						
Quick DASH	59.46	24.32	16.22						
**Pain on activity/loading**									
Tennis Elbow Functional Scale	10.81	72.97*	16.22	18.92	81.08				
PRTEE	64.86	18.92	16.22	83.78	16.22				Items in PRTEE-pain too specific, may not cover everyone. Gripping nominated as main provocative movement
**Function**									
PRTEE	64.86	13.51	21.62	89.19	10.81				Some items in PRTEE-function may not represent their experience

Insufficient psychometric evidence	In COS-LET?			Interim Suggestion?		UK Focus Group (n=3/5)	UK Comments	AUS Focus Group (n=2/4)	AUS comments
In COS-LET	**Yes**	**No**	**Unsure**	**Yes**	**No**				
Interim suggestion									
Interim suggestion - patients									
Unsure									
Not in COS-LET									
**Pain over a specified timeframe**									
Tennis Elbow Functional Scale	8.11	72.97*	18.92	24.32	75.68*		It was felt that this domain had been covered by PRTEE and EQ5D		Descriptors seem reasonably similar to PRTEE. Not sure how valuable would be to have a single snap shot of a day. Depends on what doing that day. PRTEE better
Proposed by expert: ?NRS or VAS about pain over specified time									
**Participation (daily activities, work, sport)**							? Covered by section B of PRTEE but only reflects the last week		
Return to sport				56.76	43.24		One not sporty, so not relevant to them		
Time off work				70.27	29.73		2 participants didn't have any time off		May not be sensitive. Could there be a qualifying question regarding occupation/physical activity involved? Changed to 'time off from daily activities' might be more applicable
Total Elbow Scoring System				10.81	89.19*				

Insufficient psychometric evidence / In COS-LET / Interim suggestion / Interim suggestion - patients / Unsure / Not in COS-LET	In COS-LET? Yes	No	Unsure	Interim Suggestion? Yes	No	UK Focus Group (n=3/5)	UK Comments	AUS Focus Group (n=2/4)	AUS comments
**Patient rating of condition**									
Global perceived effect score				35.14	64.86				
Global Rating of Change				56.76	43.24		More inclusive having words and numbers		
Patient Satisfaction Scale				45.95	54.05		Satisfaction is different to the effect of treatment		
**Physical function capacity (including strength)**									
Grip strength (maximum)	16.22	45.95	37.84	32.43	67.57		How can you accurately measure max strength if inhibited by pain?		
Pain free grip strength	40.54	29.73	29.73	64.86	35.14				
**Psychological factors**									
Hospital Anxiety and Depression Scale				37.84	62.16		Lacks relevance		A lot of items don't reflect psyc status related to the condition
Tampa Scale of Kinesophobia				43.24	56.76		Concerns regarding too many questions (TSK-17) but more condition-specific		Both felt more relevant to their condition

Insufficient psychometric evidence / In COS-LET / Interim suggestion / Interim suggestion - patients / Unsure / Not in COS-LET	**In COS-LET?**			**Interim Suggestion?**		UK Focus Group (n=3/5)	UK Comments	AUS Focus Group (n=2/4)	AUS comments
	**Yes**	**No**	**Unsure**	**Yes**	**No**				
**Quality of life**									
EQ5D				59.46	40.54				
SF-12				37.84	62.16		I see that and switch off! It just looks bad. It is an assault on the eyes! People would just glaze over		More broader and wholistic? More context when trying to answer questions. 5 vs 12 items not an issue
