# Supplementary material for: Development of a core outcome set for lateral elbow tendinopathy (COS-LET) using best available evidence and an international consensus process
Source: Br J Sports Med. 2022 Feb 8;56(12):657–66. doi: 10.1136/bjsports-2021-105044 (PMC9163713; doi:10.1136/bjsports-2021-105044)
Supplement: Supplementary data [file bjsports-2021-105044supp004.pdf]

Core Outcome Set for Lateral Elbow Tendinopathy (COS-LET) Tool 2022

Patient-rated tennis elbow evaluation:

The questions below will help us understand the amount of difficulty you have had with your arm in the past week. You will be describing your ***average*** *arm symptoms* ***over the past week*** *on a scale 0-10. Please provide an answer for all questions. If you did not perform an activity because of pain or because you were unable ,then you should circle a "10". If you are unsure please estimate to the best of your ability. Only leave items blank if you never perform that activity. Please indicate this by drawing a line completely through the question.*

1. PAIN in your affected arm	
Rate the average amount of pain in your arm ***over the past week*** *by circling the number that best describes your pain on a scale from 0-10. A* ***zero*** *(0) means that you* ***did not have any pain*** *and a* ***ten*** *(10) means that you had* ***the worst pain imaginable.***	
RATE YOUR PAIN:	No Pain ... Worst Imaginable
When your are at rest	0 1 2 3 4 5 6 7 8 9 10
When doing a task with repeated arm movement	0 1 2 3 4 5 6 7 8 9 10
When carrying a plastic bag of groceries	0 1 2 3 4 5 6 7 8 9 10
When your pain was at its least	0 1 2 3 4 5 6 7 8 9 10
When your pain was at its worst	0 1 2 3 4 5 6 7 8 9 10

Please turn the page......

Bateman M, *et al. Br J Sports Med* 2022; 56:657–666. doi: 10.1136/bjsports-2021-105044

2. FUNCTIONAL DISABILITY

A. SPECIFIC ACTIVITIES

*Rate the **amount of difficulty** you experienced performing each of the tasks listed below, over the past week, by circling the number that best describes your difficulty on a scale of 0-10. A zero (0) means you did not experience any difficulty and a **ten (10)** means it was **so difficult you were unable to do it at all**.*

	No Difficulty										Unable To Do
Turn a doorknob or key	0	1	2	3	4	5	6	7	8	9	10
Carry a grocery bag or briefcase by the handle	0	1	2	3	4	5	6	7	8	9	10
Lift a full coffee cup or glass of milk to your mouth	0	1	2	3	4	5	6	7	8	9	10
Open a jar	0	1	2	3	4	5	6	7	8	9	10
Pull up pants	0	1	2	3	4	5	6	7	8	9	10
Wring out a washcloth or wet towel	0	1	2	3	4	5	6	7	8	9	10

B. USUAL ACTIVITIES

*Rate the **amount of difficulty** you experienced performing your **usual** activities in each of the areas listed below, over the past week, by circling the number that best describes your difficulty on a scale of 0-10. By "usual activities", we mean the activities that you performed **before** you started having a problem with your arm. A **zero** (0) means you did not experience any difficulty and a **ten** (10) means it was so difficulty you were unable to do any of your usual activities.*

1. Personal activities (dressing, washing)	0	1	2	3	4	5	6	7	8	9	10
2. Household work (cleaning, maintenance)	0	1	2	3	4	5	6	7	8	9	10
3. Work (your job or everyday work)	0	1	2	3	4	5	6	7	8	9	10
4. Recreational or sporting activities	0	1	2	3	4	5	6	7	8	9	10

Comments:

© MacDermid 2005

Pain subscale total = ………… (max 50)

Function subscale total (specific activities + usual activities /2) = ………… (max 50)

Total (pain subscale + function subscale) = ………… (max 100)

Pain items 1, 4, 5 total = ………… (max 30)

Bateman M, *et al. Br J Sports Med* 2022; 56:657–666. doi: 10.1136/bjsports-2021-105044

Pain-free grip strength:

Pain-free grip is measured using a handgrip dynamometer that measures grip pressure in pounds or kilograms. This is done with the patient's shoulder in the neutral position, the elbow in 90 degrees of flexion, and the forearm in the neutral position of supination/pronation. The patient slowly squeezes the dynamometer handle until he or she feels pain at the elbow. The force generated is recorded, and the average value of 3 repeated assessments is used for data analysis.

Left Arm		Right Arm	
Measurement 1	………..	Measurement 1	………..
Measurement 2	………..	Measurement 2	………..
Measurement 3	………..	Measurement 3	………..
Mean	………..	Mean	………..

Please rate from 0-10 how much pain you have when gripping strongly.

No pain Worst imaginable

0 1 2 3 4 5 6 7 8 9 10

In the last month, how many days have you had off work due to your elbow pain?

☐ days Not applicable (I don't work) ☐

Bateman M, *et al. Br J Sports Med* 2022; 56:657–666. doi: 10.1136/bjsports-2021-105044
